# “Antenna Effect”‐Enhanced AuNPs@rGO Photothermal Coating Promotes 3D Printing of Osteogenic Active Scaffolds to Repair Bone Defects after Malignant Tumor Surgery

**DOI:** 10.1002/advs.202417346

**Published:** 2025-02-20

**Authors:** Xinyu Xu, Hao Wang, Xiaohan Mei, Dapeng Zeng, Zehao Yu, Shibo Liu, Ruiyan Li, Yanguo Qin

**Affiliations:** ^1^ Department of Orthopaedics The Second Hospital of Jilin University Jilin University Changchun 130041 P. R. China; ^2^ Department of Plastic Surgery Beijing Tsinghua Changgung Hospital School of Clinical Medicine Tsinghua University Beijing 102218 P. R. China; ^3^ Joint International Research Laboratory of Ageing Active Strategy and Bionic Health in Northeast Asia of Ministry of Education Jilin University Changchun 130041 P. R. China; ^4^ National & Local Joint Engineering Laboratory for Synthesis Technology of High‐Performance Polymer College of Chemistry Jilin University Changchun 130012 P. R. China

**Keywords:** 3D printing, bone repair, bone tumor, photothermal, polyetherimide

## Abstract

Malignant bone tumor defects are difficult to treat because of the simultaneous need for tumor treatment and bone‐repair promotion. This study presents a bioactive composite scaffold (T‐rGO@Au) for personalized bone defect repair and bone tumor treatment. The T‐rGO@Au scaffold has a porous structure, and its mechanical properties are close to those of human cancellous bone. The T‐rGO@Au scaffold can induce upregulation of osteopontin (OPN), RUNX‐2, and osteocalcin (OCN) gene expression. In vivo experiments showed that the bone volume/total volume (BV/TV) ratio with the T‐rGO@Au scaffold was the highest. The new bone was tightly integrated with the implant, demonstrating effective osseointegration. The T‐rGO@Au scaffold locally generated high temperatures and reactive oxygen species under near‐infrared excitation, and AuNPs enhanced the photothermal performance of rGO through the “antenna effect.” Furthermore, in vitro experiments showed that the tumor cell nuclei were destroyed, late‐stage apoptotic cells increased, and cell morphology was severely damaged. Additionally, RNA‐seq revealed that tumor cell destruction was mediated through signaling pathways, such as the MAPK pathway. In vivo antitumor experiments also demonstrated that the T‐rGO@Au scaffold significantly inhibited the growth of tumor cells within 2 weeks. Thus, the T‐rGO@Au scaffold provides a new treatment strategy for the development of implantable scaffolds for bone tumor defects.

## Graphical abstract

The T‐rGO@Au scaffold incorporates an rGO@AuNP composite photothermal coating inspired by the “antenna effect.” Under near‐infrared light irradiation, the scaffold induces the generation of reactive oxygen species (ROS) and localized thermal stimulation, leading to the effective elimination of tumor cells. Furthermore, osteogenic components, including rGO, AuNPs, and β‐TCP, promote bone regeneration under pathological conditions.

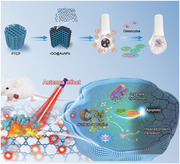

## 1 Introduction

Osteosarcoma (OS) is a common primary malignant bone tumor exhibiting strong invasiveness, rapid development, and easy metastasis.^[^ ^1^, ^2^, ^3^ ^]^ The clinical application of the treatment model of “preoperative chemotherapy–surgical resection–postoperative chemotherapy” has somewhat improved patients’ limb salvage rate and prognosis.^[^ ^4^ ^]^ However, the radical resection of OS often results in irreparable bone defects, necessitating bone grafting and reconstruction. In addition, postoperative chemotherapy often fails to effectively eliminate residual tumor cells near the surgical site and may lead to chemotherapy‐related side effects. To address this clinical problem, integrated biomaterials for bone defect filling and residual bone tumor clearance need to be fabricated for optimizing clinical OS treatment.^[^ ^5^ ^]^

Owing to the rapid development of three‐dimensional (3D) printing technology, the scaffold shape can be customized according to the bone defect, and personalized scaffolds have become the mainstream choice for mending bone defects.^[^ ^6^ ^]^ The bone scaffolds used to fill tumor bone defects are required to have both antitumor treatment and osteogenesis promotion functions.^[^ ^7^ ^]^ From the bone repair perspective, continuous attachment sites of osteoblasts and reasonable space for new bone and vascular growth need to be provided, and the promotion of osteogenesis needs to be sustained and effective.^[^ ^8^ ^]^ Polyetherimide (PEI) is a popular bone scaffold material due to its heat resistance, mechanical properties, chemical stability, and modulus close to that of the human bone tissue. 3D printing technology can be utilized for PEI bone scaffolds to adapt to irregular bone defect shapes through shape and pore customization, new bone promotion, blood vessel growth, and nutrient exchange.^[^ ^9^, ^10^, ^11^, ^12^, ^13^ ^]^ To enhance the osteogenic ability of 3D‐printed PEI scaffolds, adding inorganic molecules to promote osteogenesis is an effective method.^[^ ^14^ ^]^ Furthermore, the addition of β‐tricalcium phosphate (β‐TCP) to the polymer material can induce novel bone formation and promote the normal growth of blood vessels and bone tissue.^[^ ^15^ ^]^ Therefore, a 3D‐printed PEI composite β‐TCP scaffold could be used for treating tumor bone defects.

From the antitumor perspective, antitumor ability needs to be continuously provided after operation to prevent bone tumor recurrence.^[^ ^16^ ^]^ Photothermal therapy (PTT) is a novel noninvasive tumor treatment method that converts light energy into heat energy to induce tumor cell death.^[^ ^17^ ^]^ Advantageously, it exhibits high efficiency, good spatial specificity, and easy remote control.^[^ ^18^, ^19^, ^20^ ^]^ Through laser irradiation control, the treatment scope can be concentrated, the thermotherapy area can be limited, and the damage to other organs and tissues can be reduced.^[^ ^18^, ^21^ ^]^ The rational design of efficient photothermal agents with excellent photothermal conversion properties is an important topic.^[^ ^22^ ^]^ Gold nanoparticles (AuNPs) and reduced graphene oxide (rGO) play an essential role in bone‐tumor‐related biomaterials and are widely used as photothermal agents.^[^ ^23^ ^]^ AuNPs and rGO can be combined through electrostatic interactions, with AuNPs acting as “local nanoantennas,” which enhance light absorption by concentrating near‐infrared (NIR) light energy at their surface, significantly improving the NIR absorption of rGO compared to pure graphene oxide.^[^ ^24^ ^]^ The rGO@AuNP composite photothermal agent constructed based on the “antenna effect” of AuNPs can yield higher heat at lower power and increase the photothermal treatment effect. Furthermore, rGO and AuNP could lead to the production of reactive oxygen species (ROS) in tumor cells under NIR stimulation, thus enhancing the tumor therapy effect.^[^ ^25^, ^26^ ^]^ Additionally, rGO and AuNPs have been proven to have certain osteogenic potential and can somewhat promote bone repair.^[^ ^27^, ^28^ ^]^ From the neoplastic bone defect perspective, the combination of PTT and 3D‐printed PEI/β‐TCP scaffold is highly advantageous.

**Figure 1:**
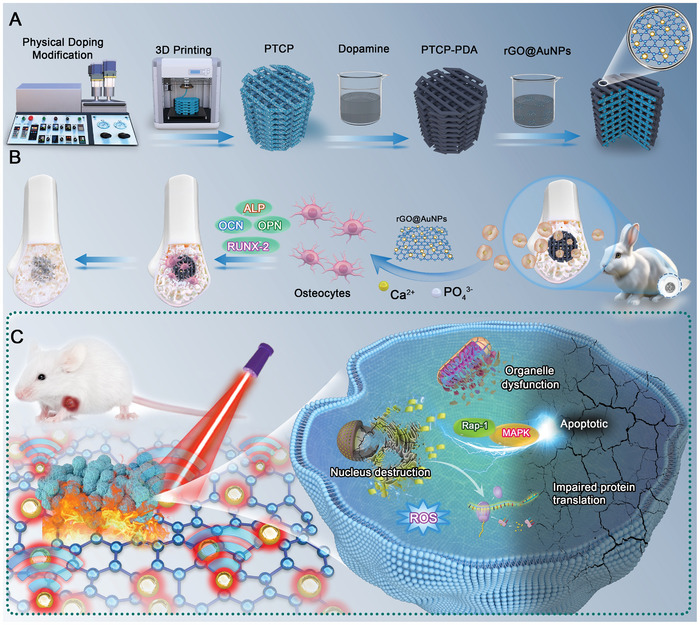
Schematic diagram of T‐rGO@Au scaffold for the treatment of neoplastic bone defect. A) Preparation of T‐rGO@Au composite scaffolds via a twin‐screw extruder, 3D printer, and PDA surface modification technology. B) Application of the T‐rGO@Au scaffold to repair femoral condylar bone defects in rabbits. C) The “antenna effect” enhances the photothermal performance of the T‐rGO@Au scaffold, generating heat and ROS to induce tumor cell damage and apoptosis.

Herein, a PEI/β‐TCP porous scaffold was prepared through 3D printing, and an rGO@AuNPs coating was loaded on the scaffold surface, yielding a T‐rGO@Au scaffold. Then, the physical and chemical properties of the scaffold were characterized, and the in vitro biocompatibility, bone formation promotion ability, and in vivo osseointegration promotion ability were verified. After characterizing the photothermal properties of the scaffold, its ability and mechanism to induce tumor cell damage in vitro were examined, and its antitumor efficacy was verified through in vivo experiments. Furthermore, RNA‐seq was conducted, and the potential mechanisms underlying tumor suppression were investigated through bioinformatics analysis. The constructed 3D‐printed T‐rGO@Au scaffold has two functions, i.e., eliminating tumor cells and repairing bone tissue, which can solve the two key problems of bone defect and residual tumor tissue. **Figure** **1** depicts the schematic of this study.

## 2 Experimental Section

### 2.1 Fabrication of PEI/β‐TCP Scaffold

#### 2.1.1 Preparation of PEI Filaments

Biograde PEI particles (Sigma‐Aldrich Co., USA) were first dried in an oven at 120 °C for 2 h. After drying, the particles were crushed using a crushing mixer (Ruian Yongli Pharmaceutical Machinery Co., China) to produce PEI powder. Next, melt extrusion was performed using a twin‐screw extruder (Wuhan Ruiming Co., China) with the parameters set as outlined in Table S1 (Supporting Information), followed by preheating. The PEI powder was fed into the extruder's feed port to initiate the extrusion process. The filaments' diameters were measured accurately with a laser diameter gauge. The filaments were then dried at 60 °C for 24 h before being input into a 3D printer.

#### 2.1.2 Preparation of PEI/β‐TCP Filaments

For the PEI/β‐TCP consumables used in this study, PEI particles were first dried in an oven at 120 °C for 2 h. After drying, β‐TCP powder (Aladdin Co., Ltd., China) and PEI particles were mixed at a weight ratio of 1:9. The PEI particles and β‐TCP powder were then uniformly crushed and mixed using a crushing mixer to produce PEI/β‐TCP powder. Next, melt extrusion was performed, with the parameters set as shown in Table S1 (Supporting Information) and the system preheated. The filament was extruded, dried at 60 °C for 24 h, and subsequently fed into the 3D printer for operation.

#### 2.1.3 Scaffold Modeling and 3D Printing

The scaffold models for in vitro experiments were 10 mm × 10 mm × 3 mm cubes, while in vivo scaffolds were cylindrical with a diameter of 6 mm and a height of 9 mm. After modeling, the design was exported as an “STL” format file and sliced using Simplify3D software (Simplify3D Co., Ltd., USA). The scaffold porosity was set to 65%, with a layer thickness of 0.2 mm, a printing speed of 2000 mm min^−1^, fan speed at 30%, and a heated bed temperature set to 200 °C. For pure PEI filament, the nozzle temperature was set to 380 °C, while for PEI/β‐TCP filament, the nozzle temperature was set to 390 °C to ensure sufficient fluidity during printing. Once the printing was complete, the support structures were removed, and the scaffold was cleaned three times with ultrapure water. It was then sterilized using high‐pressure steam sterilization.

### 2.2 Surface Modification

First, deionized water was used to ultrasonic clean the PTCP scaffold twice, each time for 20 min, and the oven to fully dry. Then, weigh TRIS hydrochloride (Aladdin Co., Ltd., China) prepare the Tris–HCl buffer solution with a concentration of 10 mM and adjust the pH of the solution to 8.5. Dopamine hydrochloride (Aladdin Co., Ltd., China) was dissolved in Tris–HCl buffer solution at the concentration of 2 mg mL^−1^. The 3D‐printed PTCP scaffold was placed in a 24‐well plate (Corning Co., Ltd., USA), and the above solution was added. Under the condition of ventilation, dopamine self‐polymerized into polydopamine and adhered to the scaffolds after 12 h of light avoidance reaction. Rinse thoroughly with deionized water to obtain 3D‐printed PTCP scaffolds coated with PDA.

Then, the timing of scaffold surface modification was screened. The rGO@AuNPs were ultrasonically dispersed with *N*‐methyl pyrrolidone (NMP, Sigma‐Aldrich Co., Ltd., USA) 7 days in advance, with a concentration of 2 mg mL^−1^. The PDA‐coated PTCP scaffold was set into it and soaked for the 60s, then the scaffold was thoroughly washed with double distilled water under an ultrasonic environment, repeating this step 1–5 times (60, 120, 180, 240, and 300 s). Finally, the scaffold is thoroughly cleaned to remove NMP and unbound rGO@AuNPs. The scaffold was fully dry in the oven. The scaffolds with modification times of 60, 120, 180, 240, and 300 s were obtained, and 8 × 10^3^ MC3T3‐E1 cells were planted respectively. After incubation for 4 h, a CCK‐8 kit (Beyotime Biotechnology, Co., Ltd., China) was used to detect the cell activity. The modification time of the scaffold with the highest cell activity was selected as the modification time used in the follow‐up experiment. The scaffolds with surface‐modified rGO were recorded as the T‐rGO group, and the scaffolds with surface‐modified rGO@AuNPs were recorded as the T‐rGO@Au group.

### 2.3 Characterization

The chemical composition analysis of samples was realized by the Kratos Axis Ultra DLD X‐ray photoelectron spectroscopy system (XPS, Kratos Co., Ltd., UK). After platinum was sprayed on the scaffold surface, the elemental composition of the scaffold surface was analyzed by an X‐MAX50 energy dispersive X‐ray spectrometer (EDS, Kratos Co., Ltd., UK). Through the HORIBA Scientific LabRAM HR Evolution Raman spectrometer (HORIBA Co., Ltd., France), the analysis of rGO was carried out. After platinum was sprayed on the surface of the scaffold, the structure and surface of different scaffolds were observed by FEI Nova NanoSEM 450 Microscope (FEI Co., Ltd., USA). The Kruss DSA25 device (Kruss Co., Ltd., Germany) was used to measure the water contact angle. The mechanical properties of scaffolds were tested by the Instron5869 electronic universal testing machine (Instron Co., Ltd., America). The compressive strength, compression modulus, and stress‐strain curves are obtained from the recorded loads. Before the pressure measuring element was set at 10 kN, and the speed of the crosshead was set as 1 mm/min, one pound was preloaded. In this study, a 10% strain was selected to calculate the compressive strength of the scaffold, and a linear segment was selected to calculate the modulus of the scaffold.

### 2.4 Cell Culture and Sample Sterilization

In vitro cell experiments were carried out on ultra‐clean workbench. MC3T3‐E1 cells were used in in vitro experiments related to biocompatibility and osteogenesis. K7M2‐wt cells were used in antitumor experiments in vitro. The conditions of cell culture are 37.0 °C and 5% carbon dioxide. The cells were cultured in a Gibio high glucose medium containing 10% fetal bovine serum and 1% penicillin‐streptomycin (ThermoFisher Co., Ltd., USA). The culture medium was changed every day, and the experiment was performed after the cells reached 80–90% confluence.

### 2.5 In Vitro Examination of Biocompatibility

#### 2.5.1 Live/Dead Staining

MC3T3‐E1 cell suspension (600 µL) was seeded on different samples in 24‐well plates at the density of 1×10^4^ cells mL^−1^. After 3 and 5 days of culture, each sample was taken out and put into a new orifice plate and washed with PBS three times. After the working solution in the Calcein‐AM/PI cell double staining kit (Dojindo Co., Ltd., Japan) was mixed at room temperature, it was added to the 24‐well plate to incubate 15min without light. Then, samples were observed by Olympus BX51TF scanning fluorescence microscope (Olympus Co., Ltd., Japan). ImageJ was used to semiquantitatively analyze the cell number and mean fluorescence intensity.

#### 2.5.2 Cell Nuclear Staining Experiments

MC3T3‐E1 cell suspension (600 µL) was implanted on different samples in 24‐well plates at the density of 4 × 10^4^ cells mL^−1^. After 1 and 4 h of culture, each sample was taken out, and washed gently with PBS three times, and then was fixed with 4% paraformaldehyde for 15 min. After rinsing gently with PBS three times, each sample was stained with DAPI under the condition of avoiding light, rinsed gently with PBS again three times, and then observed by fluorescence microscope.

#### 2.5.3 Cell Scanning Electron Microscopy Experiments

MC3T3‐E1 cell suspension (600 µL) was implanted on different samples in 24‐well plates at the density of 2 × 10^4^ cells mL^−1^. After 3 days of culture, the samples were washed with PBS and fixed with 2.5% glutaraldehyde at 4 °C for 12 h. Each sample was then dehydrated with a series of gradient alcohols and air‐dried. Each sample was treated with gold spray before being observed by an XL‐30 scanning electron microscope (Philips Co., Ltd., Netherlands). ImageJ software was used for semiquantitative statistical analysis of cell numbers.

### 2.6 In Vitro Examination of Osteogenic Differentiation

MC3T3‐E1 cell suspension (600 µL) was planted on different samples in 24‐well plates at the density of 1×10^4^ cells mL^−1^. After the cells adhered, the culture medium was changed into the osteogenic induction medium. The osteogenic induction medium was obtained by adding penicillin‐streptomycin of 5 and 50 mL of fetal bovine serum to the high glucose culture medium of 450 mL. Then weigh and add vitamin C (0.2 mM), dexamethasone (10^−8^ m), and β‐glycerophosphate (10 mM). The osteogenic induction solution was changed every 24 h. After being treated with an osteogenic medium for 4 and 7 days, alkaline phosphatase (ALP) staining was performed, and the activity of ALP was quantitatively determined. After being treated with osteogenic medium for 7 and 14 days, the cells were stained with alizarin red (ARS). Following are the specific steps.

#### 2.6.1 ALP Staining

At the corresponding time point, the osteogenic induction medium was removed, each sample washed with PBS, and then fixed with paraformaldehyde for 10 min. Samples were cleaned again with PBS. Configure the ALP dyeing working solution according to the instructions of the BCIP/NBT Alkaline phosphatase chromogenic kit (Beyotime Biotechnology, Co., Ltd., China). The ALP dyeing working solution was added to the wells of each sample. The samples were incubated at room temperature in the dark for 24 h. The samples were washed three times with PBS, then observed and photographed with the SZX16 stereoscopic microscope (Olympus Co., Ltd., Japan).

#### 2.6.2 Quantitative Analysis of ALP Activity

At the corresponding time point, the osteogenic induction medium was removed, and each sample was washed with PBS. RIPA lysate (Beyotime Biotechnology, Co., Ltd., China) was used to lyse the cells adhered to the scaffold. After the cracking process is fully carried out, the supernatant is obtained by centrifugation. The alkaline phosphatase analysis kit (Beyotime Biotechnology, Co., Ltd., China) and BCA analysis kit (Beyotime Biotechnology, Co., Ltd., China) are used to measure ALP and total protein concentration. After incubation at 37 °C, the concentrations of total protein and alkaline phosphatase were measured at 405 and 562 nm using a multifunctional microplate reader (Varioskan Flash, ThermoFisher Co., Ltd., USA). The ALP activity is calculated using the following formula: ALP activity (nmol/min/mg) = (ALP concentration/ incubation time) / (total protein concentration/incubation time).

#### 2.6.3 ARS Staining

First, at the corresponding time point, the osteogenic induction medium was removed, and each sample was washed with PBS and fixed with paraformaldehyde for 10 min. After fixation is complete, wash off residual paraformaldehyde with PBS. Then, ARS staining solution (Cyagen Biosciences Co., Ltd., China) was added to each sample. The samples were incubated with the dye solution for 10 min. Finally, the samples were washed with PBS, observed, and photographed with the SZX16 stereoscopic microscope (Olympus Co., Ltd., Japan).

#### 2.6.4 Gene Expression

OCN, OPN, and RUNX‐2 gene expressions were measured by real‐time quantitative PCR, with β‐actin used as the housekeeping gene. Primer sequences are provided in Table S2 (Supporting Information). The RNA of cells from each sample was extracted using the Column Animal RNA Out Kit (Tiandz, Co., Ltd., China), and RNA concentration was measured using a micro‐spectrophotometer (NanoDrop 2000, Thermo Scientific, USA). The total RNA amount of each sample was adjusted to 200 ng, and the working solution was prepared in an EP tube. After briefly centrifuging the mixture, reverse transcription was carried out using the SureScript™ First‐Strand cDNA Synthesis Kit (Gene Fusion, USA), following the provided protocol. The reverse transcription program was set according to the kit instructions. After reverse transcription, real‐time PCR was performed using Blaze Taq SYBR Green qPCR Mix 2.0 (GeneCopoeia, USA) according to the manufacturer's guidelines. A total of 2 µL of cDNA sample was added to each well. The final reaction mixture was prepared with the following ratio: primer F (2), primer R (2), 5× BlazeTaq qPCR Mix (4), and enzyme‐free double‐distilled water (10). After thorough mixing, PCR analysis was performed using a LightCycler 480 system (LightCycler480, Roche, Switzerland), and gene expression was determined by the CT values (refer to Table S3 for details, Supporting Information).

### 2.7 In Vivo Bone Repair Effect of Scaffolds Implanted in Bone Defect of the Femoral Condyle in Rabbits

The animal experiments in this study were approved by the Ethics Committee of Changchun Longsheng Experimental Animal Technology Co., Ltd (approval number: CCLSLL‐2021012001). This section of the study utilized a rabbit femoral condyle defect implantation model to evaluate the ability of different scaffolds to promote osseointegration in vivo.

#### 2.7.1 Animal Preparation and Surgical Procedure

Experimental rabbits weighing ≈3.0 kg were selected and acclimatized for one week. Prior to surgery, the rabbits were fasted and deprived of water for 12 h. Anesthesia was induced with an intramuscular injection of 0.2 mL kg^−1^ Antai injection (DMK Biotechnology, China). Once the rabbits were fully anesthetized, local anesthesia was applied with lidocaine injection.

#### 2.7.2 Surgical Site Preparation

The surgical area around the femoral condyle was shaved using a pet shaver, covering ≈10 cm. The site was then disinfected with iodine tincture, repeated three times. An incision was made along the long axis of the femur based on the femoral condyle bone landmarks. Tissue was carefully separated layer by layer to fully expose the femoral condyle bone surface.

#### 2.7.3 Implantation Procedure

A presterilized 6‐mm surgical drill was used to create a hole at the femoral condyle implantation site, with a depth of 8–9 mm. During the drilling process, physiological saline was continuously dripped to prevent overheating, and bone debris and blood clots in the bone defect and surrounding tissues were carefully cleaned. The scaffold was then implanted into the prepared bone defect.

#### 2.7.4 Postoperative Care

After the implantation, the surgical site was disinfected, and the incision was closed with layered sutures. Following recovery from anesthesia, the rabbits were placed in cages and monitored. To prevent postoperative infection, gentamycin sulfate was injected for three consecutive days postsurgery.

#### 2.7.5 Euthanasia and Sample Collection

At 4‐ and 12‐week postimplantation, the rabbits were euthanized using an overdose of anesthetic. Bone samples from the implantation sites were harvested and fixed in 4% paraformaldehyde solution for further analysis.

#### 2.7.6 Micro‐CT Scanning and Image Analysis

Bone tissue specimens were scanned using a Micro CT (µCT50, SCANCO, Switzerland). The scanning parameters were set as follows: X‐ray tube voltage of 70 kVp, current of 200 µA, and exposure time of 300 ms. The output files were saved in DICOM format. Two‐dimensional images and three‐dimensional reconstruction images were obtained using RadiAnt DICOM Viewer (64‐bit). Quantitative analysis of the bone tissue was conducted using CT Analyser software.

#### 2.7.7 Hard Tissue Sectioning and VG Staining

After micro‐CT scanning, the specimens were processed for hard tissue sectioning using a Leica SP1600 hard tissue slicer (Leica, Germany). The sections were then subjected to VG staining.

#### 2.7.8 Quantification of Implant–Bone Integration

The implant‐of‐bone integration rate (BIC) was calculated by performing statistical analysis on the images using ImageJ software.

### 2.8 Photothermal Properties of Scaffolds

Firstly, a 0.2 W cm^−2^ near‐infrared 808nm laser was used to characterize the photothermal effect of the stent in a dry state. At the same time, an infrared thermal imager was used to record the temperature. When the light spot stabilized at the center of the sample, the timing began. Photos were taken every 2 min, and the temperature information in the infrared thermal images was plotted as a line graph to preliminarily determine the photothermal effect of the stent for subsequent research.

Since subsequent cell experiments were all carried out in a liquid environment, the power density of the scaffold was screened in the liquid environment. The power densities were 0.5, 1.0, 1.5, and 2.0 W cm^−2^, respectively. The timing started after the light spot stabilized at the center of the sample, and the temperature was recorded every 30 seconds and plotted as a line graph.

### 2.9 In Vitro PTT Antitumor Assay

K7M2‐wt cells were inoculated in a 24‐well plate at a density of 2 × 10^4^/well (600 µL). After 24 h of incubation, scaffolds were put into cell culture plates and labeled as PPT group and non‐PTT group. Among them, the PPT group was treated with 2 W cm^−2^ near‐infrared light. After 24 h, cells were stained with a Calcein‐AM/PI cell double staining kit (Dojindo Co., Ltd., Japan). At the same time, the cell viability was quantitatively detected by a CCK‐8 kit (Dojindo Co., Ltd., Japan).

Further studies were undertaken to examine the possible mechanism of tumor cell damage. K7M2‐wt cells were seeded in a 24‐well plate with a density of 3 × 10^4^/well (600 µL). After culturing for 48 h, the scaffolds were placed and treated with PTT. After the cells were treated with PPT for 24 h, the cells were stained with a reactive oxygen species detection kit (Beyotime Biotechnology, Co., Ltd., China) and Hoechst staining (Beyotime Biotechnology, Co., Ltd., China), and observed and photographed with a scanning fluorescence microscope (Olympus Co., Ltd., Japan). Semiquantitative analysis was performed on the ROS staining samples in three random fields of view to determine the average fluorescence intensity. At the same time, semiquantitative analysis was performed on the Hoechst staining samples in three random fields of view to calculate the cell number and average fluorescence intensity. K7M2‐wt cells were seeded in 24 well plates at a density of 6 × 10^4^/well (600 µL). After culturing for 24 h, they were placed in scaffolds and subjected to photothermal treatment. After 24 h, the cells were digested with EDTA‐free trypsin and treated with AnnexinV‐FITC/PI apoptosis kit (BD biosciences Co., Ltd. USA). Then, the proportion of apoptosis and necrosis was detected by flow cytometry to explore the possible mechanism of tumor damaging. K7M2‐wt cells were seeded in cell climbing pieces in 24‐well plates at a density of 6 × 10^4^/well (600 µL). After 24 h, the scaffolds were placed and subjected to photothermal treatment. After continuing to culture for 24 h, the cell morphology was observed by SEM (Philips Co., Ltd., Netherlands).

### 2.10 RNA‐seq

To explore the molecular mechanism of the photothermal effect on tumor cells, the RNA of tumor cells was determined by transcriptome sequencing and bioinformatics analysis was carried out. K7M2‐wt cells were inoculated in a 24‐well plate with a density of 1 × 10^5^/well (600 µL). After 24 h, photothermal treatment was performed. The cells were treated with Trizol reagent, and the extracted RNA was placed in liquid nitrogen for 10 min and then stored at –80 °C.

RNA sequencing analysis was performed on PEI and T‐rGO@Au groups by BGI Genomics Co., Ltd (China). Libraries were constructed using a total of 200 ng RNA and sequenced using an Agilent 2100 Bioanalyze. An average of 6.69G data was generated for each sample, and differential expression analysis was performed using the DESeq2 software package, and genes with a q value < 0.05 were considered differentially expressed. Enrichment analysis of upregulated DEGs was performed on Metascape.

### 2.11 In Vivo Antitumor Assay

The animal experiments in this part of the study were approved by the Ethics Committee of Jilin University School of Basic Medicine (2022‐154). Prepare a K7M2‐wt cell suspension with a concentration of 5 × 10^7^ mL^−1^ in advance on the ultra‐clean bench. Then, 100 µL of cell suspension was slowly injected into the armpit of BALBC mice (6–8 weeks, purchased from Beijing Vital River Laboratory Animal Technology Co., Ltd.). When the tumor volume is in the range of 300–500 mm^3^, implantation surgery can be performed. The size of implants is 1.75 mm × 1.75 mm × 5 mm. The surgical procedure is as follows. The mouse is anesthetized, the skin is prepared, and the skin on the surface of the tumor is cut by tissue cut. Then, the sterilized implant was implanted into the center of the tumor and the surface skin was sutured.

The infrared laser irradiation of 808 nm was carried out with a power density of 2 W cm^−2^, and the photothermal efficiency was explored in the tumor model of BALB/C mice, and the line chart was drawn. For the antitumor experiment, the Control, PTCP, T‐rGO, and T‐rGO@Au groups received photothermal irradiation treatment on postoperative days 2, 6, and 10. The T‐rGO and T‐rGO@Au groups were respectively set up as control groups without photothermal treatment, labeled T‐rGO NIR‐ and T‐rGO@Au NIR‐. The tumor volume was recorded every 2 days. Fourteen days later, blood samples were taken to measure blood biochemical indexes. The tumor, heart, liver, spleen, lung, and kidney were sectioned and stained.

### 2.12 Statistical Analysis

Statistical analysis was performed using GraphPad Prism 8. When comparing between two groups, an unpaired t test was used. When two normally distributed data sets have unequal variances, use the Welch unequal variance *t*‐test. When comparing more than three groups, ANOVA followed by Tukey's multiple comparison test was used. All data are expressed as mean ± SEM, and *p* values are indicated in the figures to indicate statistical significance (*p < 0.05, **p < 0.01, ***p < 0.001).

## 3 Results and discussion

### 3.1 Characterization

**Figure 2:**
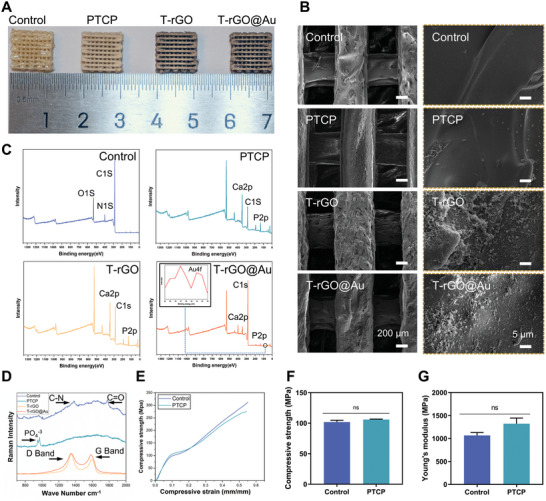
Physical and chemical characterization of samples. A) Appearance of scaffolds: Images of the scaffolds’ surface show that T‐rGO and T‐rGO@Au scaffolds have darker colors, indicating successful surface modification. B) Scanning electron microscopy (SEM)—*Left column*: Low‐magnification SEM images of the internal microstructure of the scaffolds; *Right column*: High‐magnification SEM images showing the surface morphology of each scaffold. Deposits are visible on the surface of the T‐rGO and T‐rGO@Au scaffolds. C) X‐ray photoelectron spectroscopy (XPS): Elemental composition analysis reveals the presence of Ca and P signals in PTCP, T‐rGO, and T‐rGO@Au scaffolds, with Au4f peaks detected in T‐rGO@Au scaffolds. D) Raman spectroscopy: T‐rGO and T‐rGO@Au scaffolds show clear graphene characteristic peaks, including the D band and G band. E) *Compressive stress–strain curve*: Comparison of stress‐strain curves for the control and PTCP scaffolds under compressive load. F) *Compressive strength*: The difference in compressive strength between the control and PTCP scaffolds was small and not statistically significant. G) *Young's modulus*: No significant difference in Young's modulus was observed between the control and PTCP scaffolds.

**Figure** **2**A displays the optical appearance after the preparation of the scaffolds. According to the CCK‐8 test results (Figure S1, Supporting Information), the surface modification time was selected as 240 s, where the cell viability was high. After the completion of the surface modification, the loading of rGO or rGO–AuNPs deepened the PTCP scaffold color, with T‐rGO@Au exhibiting a certain metallic luster. The above results show that rGO and rGO–AuNPs were successfully loaded on the PTCP scaffold surface. As depicted in Figure S2 (Supporting Information), the water contact angles of the control group, PTCP group, T‐rGO group, and T‐rGO@Au group were 93.4° ± 3.8°, 85.7° ± 2.6°, 66.2° ± 1.7° and 63.9° ± 0.5°, respectively. The water contact angle of the PTCP group was lower than that of the control group, which could be attributed to β‐TCP incorporation. When rGO or rGO@AuNPs were loaded on the surface by PDA, the water contact angle significantly decreased, mainly because PDA significantly increases the surface hydrophilicity. The above results show that the scaffolds of the T‐rGO and T‐rGO@Au groups exhibit better hydrophilicity than those of the control group.

To explore the effects of the internal composite and surface modification on the scaffold surface morphology, SEM was used to characterize the scaffold morphology. As presented in Figure 2B, all scaffolds displayed similar porous structures. The surface roughness of PTCP scaffolds was reduced, which could be due to the incorporation of β‐TCP for improving the 3D printing quality, and the surface still comprised relatively small pores, which were produced in the 3D printing process. The scaffold roughness improved after the loading of rGO or rGO@AuNPs as they were deposited on the scaffold surface, which improved the adherence of the cells to the scaffold surface, thus facilitating the subsequent processes of growth, migration, and osteogenic differentiation.

To ascertain the success of the internal composite and surface modification, the chemical compositions of the samples were analyzed. First, XPS was used to analyze the element composition. The C1s, Au4f, P2p, Ca2p, N1s, and O1s peaks were located at 297.98–279.18, 98.98–79.18, 143.98–124.18, 359.98–340.18, 409.98–392.18, and 544.98–525.18 eV, respectively. Figure 2C displays the XPS spectra. The control group (PEI) comprised C, N, and O, and the C1s, N1s, and O1s peaks were observed. The PTCP group comprised not only C1s, N1s, and O1s peaks but also Ca2p and P2p peaks. The T‐rGO group exhibited peaks like the PTCP group. Due to the addition of AuNP in the T‐rGO@Au group, double Au4f peaks at 84 and 88 eV were observed. The above results highlight the successful introduction of β‐TCP and AuNP, and the rGO and AuNP combination also basically verifies the rGO introduction. To analyze distribution of elemental composition on the sample surface, EDS elemental mapping was performed. As shown in Figure S3 (Supporting Information), only C, N, and O elements were present on the control group surface, which PEI already comprises. Ca and P elements were introduced into the PTCP group after the composite β‐TCP was added, which is consistent with the XPS detection results. No new elements were observed after the introduction of rGO on the surface. No difference existed between the surface element distributions of the T‐rGO and PTCP samples. After AuNP was introduced, Au elements were uniformly distributed on the sample surface, signifying that the employed surface modification method can evenly load rGO@AuNPs on the scaffold surface.

To analyze the chemical composition and conformation of the samples, Raman spectra were used to detect each group of samples. As shown in Figure 2D, the control group exhibited the characteristic peak in the 1326–1417 cm^−1^ (C–N) range and a peak at 1780 cm^−1^ (C═O), denoting the chemical composition and binding mode of the PEI elements. After the doping of β‐TCP, an obvious PO_4_^3−^ peak was observed in the 949–1087 cm^−1^ range. In the T‐rGO and T‐rGO@Au groups, the characteristic bimodal structure of the D and G bands in the 1200–1700 cm^−1^ range range confirmed the successful rGO introduction. Combining these results with the XPS detection results, the successful introduction of β‐TCP, rGO, and AuNP can be inferred, marking the fabrication of the 3D‐printed porous T‐rGO@Au scaffolds.

The coating adhesion and durability are of great significance for in vivo applications. To test the coating durability, the coating was damaged at different times (5, 10, and 30 min) using an ultrasonic cleaner (Figure S4, Supporting Information). After the longest ultrasonic treatment (30 min), high‐magnification SEM images showed that the coating was still intact, indicating that the coating exhibited good durability on the stent surface. Previous studies proved that PDA coating modification has no effect on its mechanical properties due to the good chemical and mechanical stabilities of PTCP.^[^ ^29^ ^]^ Therefore, this study uses the control and PTCP groups to explore the mechanical properties of the PEI scaffolds mixed with β‐TCP. Figure 2E,G presents the test results of mechanical properties. The stress–strain curve shown in Figure 2E denotes that after the β‐TCP addition, before reaching the maximum mechanical strength, the stress‐bearing capacity of the PTCP group scaffold is slightly higher than that of the control group scaffold under the same strain condition. The mechanical strength was calculated after the strain reached 10% (Figure 2F). Compared to the control group, the mechanical strength of the PTCP group was slightly higher than that of the control group but with no statistical difference. In terms of modulus (Figure 2G), the elastic moduli of the control and PTCP groups were within the range of cancellous bones.

### 3.2 In Vitro Examination of Biocompatibility

**Figure 3:**
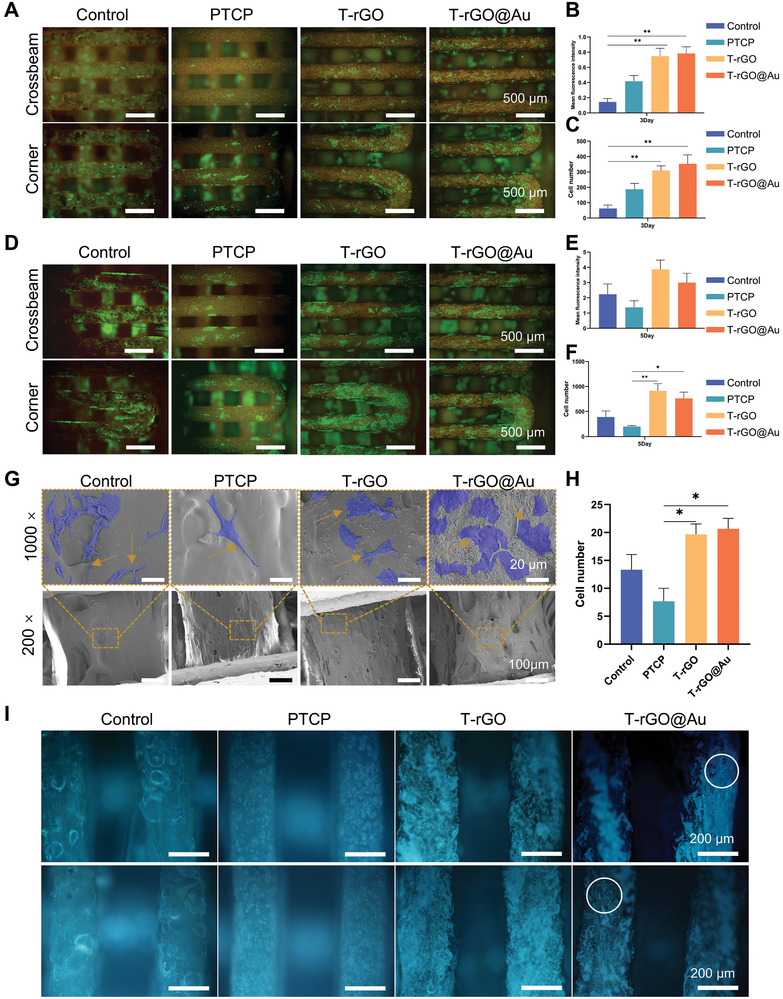
Biocompatibility evaluation of scaffolds. A–F) Fluorescence microscopy images of MC3T3‐E1 cells stained with calcein‐AM (Ca‐AM) and propidium iodide (PI) on control, PTCP, T‐rGO, and T‐rGO@Au scaffolds after 3 and 5 days of culture. T‐rGO@Au scaffolds exhibited better cell viability and proliferation trends at both 3 and 5 days. B) Semiquantitative analysis of average fluorescence intensity at 3 days: The fluorescence intensity in the T‐rGO and T‐rGO@Au groups was significantly higher than that in the Control group. C) Semiquantitative analysis of cell number at 3 days: The number of viable cells in the T‐rGO and T‐rGO@Au groups was significantly higher than that in the Control group. E) Semiquantitative analysis of average fluorescence intensity at 5 days: The fluorescence intensity in the T‐rGO and T‐rGO@Au groups was higher than that in the Control group, although this difference was not statistically significant. F) Semiquantitative analysis of cell number at 5 days: The number of viable cells in the T‐rGO and T‐rGO@Au groups was higher than that in the Control group, but the difference was not statistically significant. G) Cell morphology of MC3T3‐E1 cells adhered to the scaffold surface: SEM images showed that cells on the T‐rGO and T‐rGO@Au scaffolds were more stretched and tightly adhered to the scaffold surface compared to the Control group. H) Semiquantitative analysis of cell number from (G): Cell number statistics showed that the number of adhered cells on the T‐rGO and T‐rGO@Au scaffolds was significantly higher than that on the Control scaffold. I) DAPI staining of MC3T3‐E1 cells after 1 and 4 h of culture (1h: the above image, 4h: the below image).

To examine the activity, adhesion, growth, and migration of MC3T3‐E1 cells, the calcium‐AM/PI double staining method was used to determine the cell status and cell viability of scaffolds in each group after 3 and 5 days of incubation. In **Figure** **3**A, the green markers represent living cells. The cell survival rates of the control, PTCP, T‐rGO, and T‐rGO@Au groups on the scaffold were high at both 3 and 5 days. At 3 days, for the control scaffold, sporadic cell clusters were present on the beam, while at the scaffold corner, the cells had higher density, but their arrangement was uneven. The PTCP group comprised more evenly arranged cell clusters both in the crossbeam and edges of the scaffolds than the control group, and more cell clusters were present on the crossbeam of the PTCP scaffolds. The cell adhesion, migration, and growth in the PTCP group were better than those of the control group, signifying that β‐TCP incorporation improves the PEI biocompatibility. Compared to the other groups, the T‐rGO and T‐rGO@Au groups exhibited more cell numbers and more uniform distribution on the beam, which was highly evident at the corners (Figure 3C). Moreover, the cell fluorescence intensity of the T‐rGO and T‐rGO@Au groups was higher than that of the other groups, denoting better vitality (Figure 3B). At 5 days, the number of cells in the control, PTCP, T‐rGO, and T‐rGO@Au groups was significantly higher than that at 3 days due to cell proliferation and migration (Figure 3D). Among them, regardless of the location, the PTCP group had a more uniform distribution than the control group, and the cells on the beam also exhibited numerous advantages (Figure 3F). Compared to the other groups, the T‐rGO and T‐rGO‐Au groups had a higher fluorescence intensity of the cells, higher number of cells, and more compact and uniform arrangement of cells (Figure 3E). The above results showed that the scaffolds in each group displayed no obvious cytotoxicity within 5 days. Simultaneously, the T‐rGO and T‐rGO@Au groups exhibited greater cell proliferation and better cell adhesion ability and uniform migration distribution than the other groups, which could be due to the combined effects of TCP, rGO, and AuNP.

After the cells were incubated for 3 days, the morphology of cell adhesion on the scaffold was examined through SEM. As shown in Figure 3G, the cells in the control group were spherical and spindle‐shaped, with limited spreading and low cell–cell contacts. In the PTCP group, the cell shape was well spread, and the extension distance of the pseudopodia was longer, verifying that the PTCP scaffold has cell adhesion. The cells of the T‐rGO group were evenly distributed, and more cells were connected. The cells of the T‐rGO@Au group had a flat spreading state, the microfilaments of the cells extended a long distance, and connected with the other cells; additionally, the cell distribution was not only uniform but also showed a more orderly state. Furthermore, the T‐rGO@Au group showed the highest number of cell adhesion (Figure 3H). The cells in the PTCP, T‐rGO, and T‐rGO@Au groups had good spreading morphology, which could be attributed to the good biocompatibility of β‐TCP. The cells in the T‐rGO and T‐rGO@Au groups were highly scattered and orderly, which could be related to the increased adhesion of cells by rGO and AuNP.

To further determine the difference in early adhesion among the groups, DAPI staining was performed at 1 and 4 h. In Figure 3I, the blue marks denote the nucleus. The number of cells in each group increased with time, indicating that cell adhesion increased. After 1 h of culture, compared to the control group, the PTCP group had a more even distribution and had more cells. More cells were present on the T‐rGO and T‐rGO@Au scaffolds than on the other two scaffolds. After 4 h, the situation of each group of scaffolds was similar, and the cell distribution on the PTCP scaffolds was the most uniform. Cell clusters were present on the T‐rGO and T‐rGO@Au scaffolds. The above results show that all the scaffolds displayed good early adhesion properties, where the β‐TCP incorporation made the number of cell adhesion and the distribution more uniform, while the introduction of rGO and AuNP further increased the number of cell adhesion.

### 3.3 In Vitro Examination of Osteogenic Differentiation

**Figure 4:**
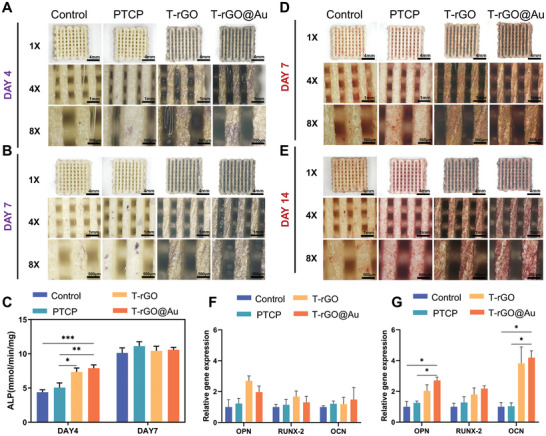
In vitro osteogenic performance evaluation. A,B) Alkaline phosphatase (ALP) staining: The T‐rGO@Au group showed significantly increased ALP activity on days 4 and 7, indicating enhanced osteogenic differentiation potential. C) Quantification of ALP activity: On day 4, the T‐rGO@Au scaffold showed the highest ALP secretion. However, this trend became insignificant by day 7. D,E) Alizarin red S (ARS) staining at 7 and 14 days: Compared with the Control, PTCP, T‐rGO, and T‐rGO@Au groups, the T‐rGO@Au group displayed denser cell distribution and stronger red staining, indicating increased mineralization. F,G) PCR analysis of osteogenic gene expression: Relative expression levels of osteogenic genes OPN, RUNX‐2, and OCN. On day 4, gene expression in the T‐rGO and T‐rGO@Au groups showed a higher trend (but not statistically significant). By day 7, compared with the Control group, the expression of OPN and OCN genes was significantly increased in the T‐rGO@Au group.

The early stage of osteogenesis mainly constitutes the proliferation and differentiation of osteoblast precursor cells and the synthesis of the extracellular matrix.^[^ ^30^, ^31^ ^]^ In this stage, osteoblast precursor cells differentiate into osteoblasts and start secreting various bioactive molecules, including key enzymes such as alkaline phosphatase (ALP). ALP is an important marker enzyme in the early stage of osteogenesis, and its activity is widely used to evaluate the biological activity and osteogenic potential of cells.^[^ ^32^ ^]^ Specifically, the increase in ALP activity is usually closely related to the early stage of osteoblast proliferation, reflecting the functionality and activity of osteoblasts in this stage.^[^ ^33^ ^]^ To detect the secretion of ALP, ALP staining was used after 4 and 7 days of incubation. In **Figure** **4**A,B, blue–purple nodules represent the secretion of ALP. In the ALP‐stained images, the density and number of bluish‐purple nodules per scaffold increased over time. Further, observe the staining of different groups of scaffolds at the same time. The number and density of purple nodules in PTCP scaffolds are more than those in Control scaffolds, which proves the effect of β‐TCP incorporation on scaffold ALP secretion. Compared with Control and PTCP scaffolds, there are more blue–purple nodules on T‐rGO and T‐rGO@Au scaffolds. Among them, the number and density of blue–purple nodules on T‐rGO@Au scaffolds were the highest, which may be attributed to the effect of rGO on osteogenesis and the additional effect of AuNP on bone formation.^[^ ^34^ ^]^ The ALP activity was quantitatively analyzed to examine the activity of ALP in cells on different scaffolds. Figure 4C displays the real‐time ALP activity of the control, PTCP, T‐rGO, and T‐rGO@Au scaffolds after 4 and 7 days of osteogenic induction. After 4 days, the real‐time activity of ALP showed a trend of T‐rGO@Au > T‐rGO > PTCP > control, wherein T‐rGO exhibited considerable statistical significance compared to PTCP. T‐rGO@Au exhibited considerable statistical significance compared to control and PTCP. After 7 days, the PTCP activity was the highest, followed by T‐rGO and T‐rGO@Au, and no significant difference existed among all the groups. Hence, β‐TCP introduction can promote the ALP activity, while the loading of rGO and AuNP greatly influences the early ALP activity, which signifies that the introduction of components such as β‐TCP promotes the early osteogenic differentiation of osteoblast precursor cells.

Late osteogenesis mainly constitutes the maturation, mineralization, and formation of a new bone of osteoblasts. This process not only contributes to the remodeling and repair of bones but also plays an essential role in maintaining the bone density, bone quality, and overall bone health.^[^ ^35^ ^]^ During this stage, mature osteoblasts form a mineralized bone matrix, and specific evaluation methods are employed, such as Alizarin red staining (ARS).^[^ ^36^ ^]^ In the ARS staining image in Figure 4D,E, the red nodules represents calcium deposition. After 7 and 14 days of culture, the number and density of the red nodules in each group increased with time. After 7 days, compared to the Control scaffolds, the PTCP, T‐rGO, and T‐rGO@Au scaffolds had higher nodule numbers and densities, and the difference among the PTCP, T‐rGO, and T‐rGO@Au scaffolds was not significant. After 14 days, the nodule number and density in each group significantly increased. Compared to the control group, the PTCP, T‐rGO, and T‐rGO@Au groups had higher nodule numbers and densities. PTCP exhibited a very uniform distribution of calcium deposition; compared to the other groups, the T‐rGO@Au group had the largest nodule density and number, followed by the T‐rGO scaffold. The above results denote that the amount of calcium deposition significantly varied among the groups on the 14th day. This illustrates that as osteogenesis progresses, osteoblast precursor cells gradually differentiate into osteoblasts and secrete calcium to form calcium nodules, which are mineralized in the cell process. β‐TCP addition significantly enhanced this phenomenon, and the introduction of rGO and AuNPs further increased the calcium deposition.^[^ ^28^, ^37^ ^]^

During the osteogenesis process, cells promote the mineralization and maturation of the bone matrix by secreting markers, including OCN and OPN, and provide support for the dynamic balance of bones.^[^ ^38^, ^39^ ^]^ Figure 4F,G depicts the gene expressions of all the groups at 4 and 7 days. OPN is an important marker of osteogenic differentiation. On the 4th day, the OPN expression of the T‐rGO and T‐rGO@Au groups was high, with T‐rGO being higher. On the 7th day, the OPN expression of the PTCP group was higher than that of the control group, and that of the T‐rGO@Au group was the highest among all the groups. RUNX‐2 plays an important role in osteoblast formation by stimulating the transcription of osteoblast differentiation genes. On the 4th day, the RUNX‐2 expression followed the following order: T‐rGO > T‐rGO@Au > PTCP > control. On the 7th day, the RUNX‐2 expression in the T‐rGO@Au group was higher than that in the other groups. Moreover, OCN is an important indicator of osteogenic differentiation. Regardless of the day, the OCN expression of the T‐rGO@Au group was the highest, followed by the T‐rGO group, and it gradually increased from the 4th to 7th day. The above results suggest that β‐TCP, rGO, and AuNP have different tendencies to enhance the gene expression in promoting osteogenic differentiation, and their combined introduction in the interior and surface of control scaffolds can lead to the promotion of bone differentiation at the gene level.

### 3.4 In Vivo Bone Repair Effect of Scaffolds Implanted in the Bone Defect of the Femoral Condyle in Rabbits

**Figure 5:**
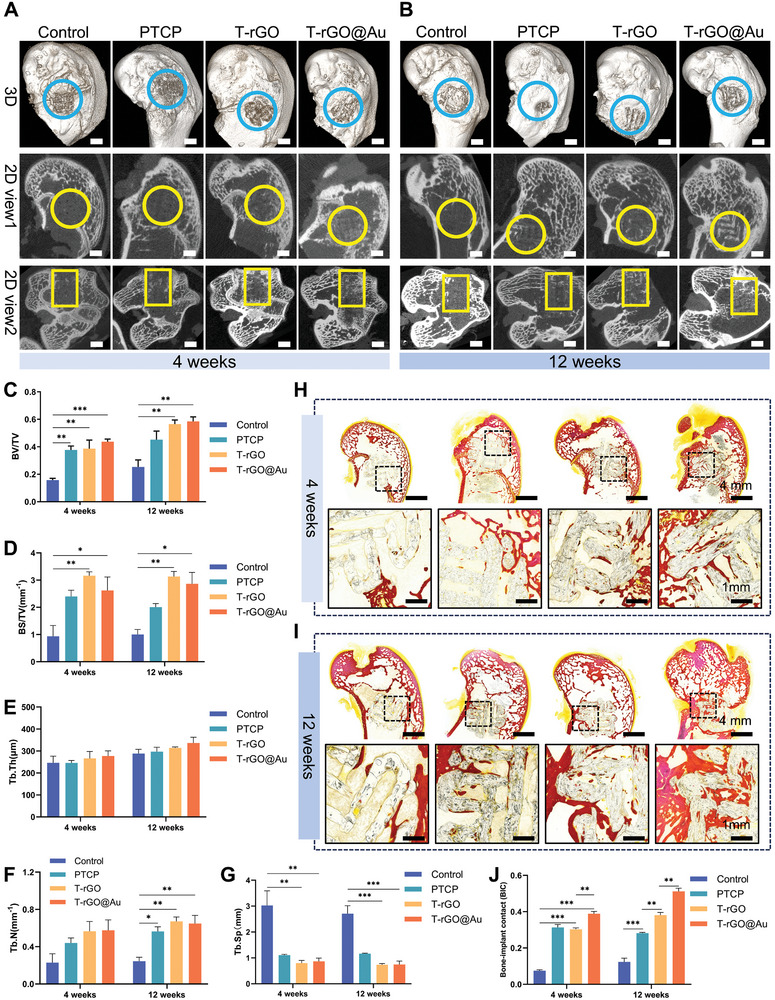
In vivo osteogenesis experiments. A,B) Micro‐CT 3D and 2D reconstruction images: Bone regeneration effects of different material groups after 4 and 12 weeks. The T‐rGO@Au group exhibited more pronounced bone tissue reconstruction and increased density at all time points. Scale bars: 2 mm. C,D) Quantitative analysis of bone parameters by Micro‐CT at 4 and 12 weeks: C) Bone volume fraction (BV/TV). D) Bone surface area to tissue volume ratio (BS/TV). E,G) Quantitative analysis of trabecular parameters by Micro‐CT at 4 and 12 weeks: E) Trabecular thickness (Tb.Th). F) Trabecular number (Tb.N). G) Trabecular separation (Tb.Sp). H,I) VG staining of bone tissue sections: In the T‐rGO@Au group, bone tissue (red) growing into the scaffold can be observed, in close contact with the scaffold surface. J) Bone‐implant contact (BIC) analysis: Semiquantitative analysis of bone‐implant contact in VG‐stained sections.

Micro‐CT imaging was conducted to compare the different samples during the repair of rabbit femoral condyle defects. **Figure** **5**A,B displays the CT reconstruction images of the scaffold site and surrounding bone tissue after 4 and 12 weeks of implantation. The 3D reconstruction showed that the bone tissue around the implanted area of the scaffold was well intact and had no fractures. As the bone repair time progressed, the implanted area was slightly covered with a new bone. The two‐dimensional (2D) reconstruction showed that the scaffold was successfully implanted into the bone, the external bone tissue was tightly surrounded, and a new bone grew inside. In the figure, the bone tissue is white, and the scaffold is light gray. The scaffold cross‐section in each group was circular, and the amount of new bone in the control group was limited, with only cord‐like new bone growth at the contact area between the scaffold and bone tissue. In the PTCP group, owing to the β‐TCP addition, the surrounding new bone formation increased. In the T‐rGO and T‐rGO@Au groups, after the rGO and AuNP addition, considerable new bone formation occurred inside the scaffold, the scaffold pores were filled, and the osteogenic properties improved. The 3D reconstruction results of the interior of the scaffold (Figure S5, Supporting Information) were consistent with the 2D image results. The control, PTCP, T‐rGO, and T‐rGO@Au groups can all promote bone integration and repair in vivo, and the PTCP, T‐rGO, and T‐rGO@Au groups exhibited ideal new bone formation at 12 weeks.

Since the 2D and 3D images do not provide quantitative information about new bone growth, the bone parameters (BV/TV and BS/TV) of the Micro‐CT of each group at 4 and 12 weeks were quantitatively analyzed to comprehensively evaluate the bone mass. The trabecular bone parameters (Tb.N, Tb.Th, and Tb.Sp) were quantitatively investigated to evaluate the bone microstructure and osteocyte activity characteristics. Figure 5C displays the BV/TV result, directly reflecting the bone mass variations. The trend of T‐rGO@Au > T‐rGO > PTCP > control was observed at both 4 and 12 weeks. BS/TV can indirectly reflect the bone mass variations (Figure 5D), and its trend supports the significant promotion of bone mass for the T‐rGO@Au scaffolds. This could be because β‐TCP, rGO, and AuNP promote osteogenesis, as mentioned earlier, and the combined use of scaffolds could improve the osteogenesis of scaffolds. Figure 5E–G presents the analysis results of the trabeculae in each group of scaffolds, reflecting the geometry, mechanical properties, and functional status of the trabeculae. Tb.Th is a parameter for evaluating the trabeculae thickness. Figure 5E shows that Tb.Th is the highest in the T‐rGO@Au group at 12 weeks. The Tb.N and BV/TV results were consistent. T‐rGO@Au significantly promoted the increase in the number of trabeculae around the implant. Tb.Sp is the average width of the medullary cavity between trabeculae, which increases during bone absorption. Tb.Sp was the smallest in the T‐rGO@Au group. The above quantitative parameters for the trabeculae illustrate that the T‐rGO@Au group has a good promoting effect on trabeculae maturation, which could be due to the higher number of active bone cells inside it.

To analyze the bone tissue growth, VG staining was performed on the bone tissue samples at 4 and 12 weeks (Figure 5H,I). At 4 weeks, the amount of internal bone growth in the control group was low, but no fibrous tissue wrapping was present on the scaffold surface, evidencing the application potential of PEI as a bone implant. Compared to the control scaffold, the PTCP scaffold had better bone growth ability, and the new bone was cord‐like and in close contact with the scaffold. Considerable new bone tissue growth was observed for the T‐rGO scaffold, and bone tissue was also observed in the internal pores. The T‐rGO@Au group showed the best bone growth, and the new striped bone was in close contact with the scaffold interior. The bone growth in each group at 12 weeks was more than that at 4 weeks. In the control group, more cord‐like bone grew in contact with the bone tissue. In the PTCP scaffolds, obvious new bone growth was observed in the pores. The new bone mass on the T‐rGO scaffold was more than that on the control and PTCP scaffolds, and the new bone was closely combined with the scaffold beam. In the T‐rGO@Au scaffold, the internal pores were almost filled with new bone tissue, the scaffold was closely combined with the new bone, the striped bone tissue was in direct contact with the scaffold beam, and the surrounding new bone tissue was closely combined with the scaffold surface. The semiquantitative statistics of the implant–bone integration rate also supported this conclusion (Figure 5J): at the implant–bone interface, the T‐rGO@Au scaffold was well integrated with the bone, highlighting its excellent bone integration effect.

In vitro and in vivo osteogenesis experiments showed that the T‐rGO@Au group scaffolds performed well in the early, late, and in vivo osteogenesis experiments, suggesting that T‐rGO@Au scaffolds are useful for bone tissue engineering applications. In bone tissue engineering scaffolds, nanocomponents play an essential role in regulating the cell behavior or cell microenvironment. Numerous studies have demonstrated that GO is a candidate material for accelerating gene expression and osteogenic differentiation.^[^ ^40^ ^]^ rGO and HAp synergistically increase the OCN expression level in MC3T3‐E1 cells.^[^ ^41^ ^]^ Specifically, in the graphene microelectrodes used for hMSC chip operation, rGO promotes the adhesion and alignment of hMSC cells and accelerates their osteogenic differentiation, which is similar to the results of this study, wherein OCN gene expression significantly increased 7 days after rGO loading. Moreover, AuNPs promote osteogenic differentiation and mineralization through gold ion release and endocytosis. AuNPs enhance cell proliferation, differentiation, calcium nodule deposition, and RUNX ‐ 2expression.^[^ ^42^ ^]^ This is consistent with the results of cell live–dead staining (Figure 3A,D), Alizarin red staining (Figure 4D,E), and PCR experiments (Figure 4F,G). Additionally, the surface modification process inevitably influences the material's hydrophilicity. The increase in hydrophilicity promotes early cell adhesion and protein adsorption and indirectly promotes cell proliferation and differentiation. However, osteogenic functional components are activated through genes, and signal regulation significantly influences osteogenic differentiation and long‐term bone tissue reconstruction, as observed through 12 weeks of BV/TV. Therefore, future scaffold designs should focus on optimizing functional components to improve bone tissue repair efficiency.

### 3.5 Photothermal Properties of Scaffolds

**Figure 6:**
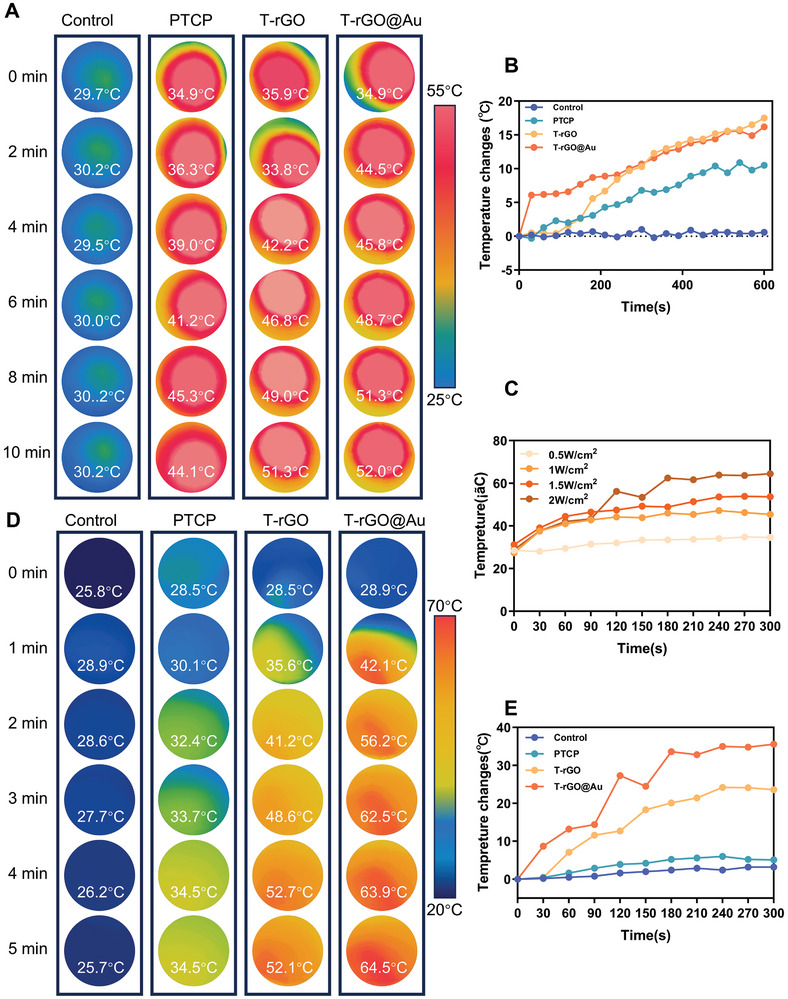
Photothermal performance evaluation of different samples under laser irradiation. A) Infrared thermal imaging: Surface temperature changes of different samples (dry) over 0–10 min under near‐infrared light irradiation with a power density of 0.2 W cm^–^^2^. The temperature of the T‐rGO@Au sample reaches ≈52 °C after 10 min. B) Temperature change curve with time: Dynamic changes in surface temperature of each sample under near‐infrared irradiation. C) Effect of laser power density on temperature change: In the power density range of 0.5–2 W cm^–^^2^, higher power densities lead to more significant temperature increases. D) Infrared thermography under high power density: Under a laser power density of 2 W cm^–^^2^, the temperature of the T‐rGO@Au sample (in a humid environment) rises to about 64.5 °C within 5 min, showing a significant temperature rise. E) Temperature change curve under high power density: At a power density of 2 W cm^–^^2^, the temperature change of the T‐rGO@Au sample is significantly higher than that of the other sample groups, confirming its superior photothermal conversion performance.

The photothermal effect of each group of brackets was tested in a dry environment. As shown in **Figure** **6**A,B, the NIR light was adjusted to 0.20 W cm^−2^. The temperature of the control group did not increase with time. PTCP group displayed a weak photothermal effect, increasing from room temperature to 44.1 °C in 10 min, which has rarely been focused on in previous studies. The temperature of the T‐rGO scaffold increased to 42.2 and 51.3 °C after 4 and 10 min under infrared irradiation, respectively. However, the T‐rGO@Au scaffolds showed excellent photothermal properties, increasing to 44.5 °C after only 2 min and 52.0 °C after 10 min. The above results signify that PTCP, T‐rGO, and T‐rGO@Au scaffolds all exhibit certain photothermal effects, with T‐rGO and T‐rGO@Au scaffolds reaching higher temperatures (more than 50.0 °C). This demonstrates the feasibility of photothermal scaffold materials for the photothermal eradication of bone tumors.

However, the follow‐up experiments were mainly conducted in a liquid environment, because liquids have large specific heat capacities and strong thermal conductivities; thus, their power densities need to be appropriately increased. Therefore, the power densities of 0.5, 1.0, 1.5, and 2.0 W cm^−2^ were used for the subsequent experiments on the T‐rGO@Au scaffolds. As depicted in Figure 6C, the treatment temperature was steadily increased and kept above 50.0 °C, which is the commonly used basic temperature of PTT under the power density of 2.0 W cm^−2^. Therefore, 2.0 W cm^−2^ was selected as the power density during the photothermal treatment herein.

Thereafter, the photothermal performance of each scaffold in a liquid environment was characterized. Simultaneously, the infrared thermal images (Figure 6D) and temperature line graphs (Figure 6E) of all the scaffold groups under 2.0 W cm^−2^ were obtained. After 10 min of NIR light irradiation, the temperature of the control scaffold in the liquid environment did not increase. However, unlike in the dry environment, the photothermal effect of PTCP in the humid environment did not significantly increase the temperature of the liquid, with only a 6 °C rise from 28.5 to 34.5 °C. The temperature of the T‐rGO scaffold increased to 41.2 °C in 2 min and 52.7 °C in 4 min and stayed above 50.0 °C. The excellent photothermal properties of the T‐rGO@Au scaffold were fully reflected, increasing to 42.1 °C within 1 min and 56.2 °C in 2 min and staying above 60.0 °C in the later stage. Stable high temperatures typically have a strong destructive effect on tumor cells. Based on the above results, compared to the T‐rGO scaffold, the photothermal performance of the T‐rGO@Au scaffold was significantly enhanced, and the maximum temperature of the T‐rGO@Au scaffold in a liquid environment reached above 60.0 °C. This could be attributed to the addition of AuNPs and the enhancement of the photothermal effect of rGO through the “antenna effect.”^[^ ^43^ ^]^

### 3.6 In Vitro PTT Antitumor Assay

**Figure 7:**
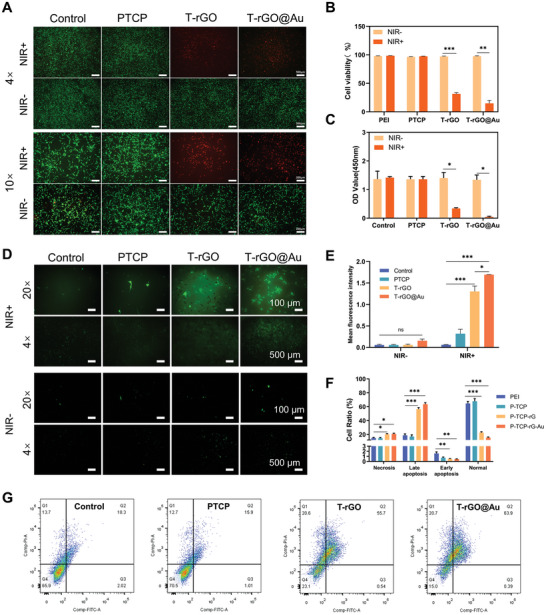
In vitro PTT antitumor assay. A,B) Calcein‐AM/PI double staining images and semiquantitative analysis (B) of K7M2‐wt cells before and after photothermal treatment. In the T‐rGO@Au group, dead cells (red) significantly increased under NIR+ conditions, indicating a strong photothermal destructive effect. The scale bars represent 500 µm at 4× magnification and 200 µm at 10× magnification. C) Cell viability of K7M2‐wt cells before and after photothermal treatment. Under NIR+ conditions, the cell viability of the T‐rGO@Au sample (OD value ≈0.2) was significantly lower than that of other samples, indicating its strong cell‐destructive effect. D,E) Detection and semiquantitative analysis of reactive oxygen species (ROS) in K7M2‐wt cells before and after photothermal treatment by fluorescence microscopy. Compared with the Control group, the T‐rGO@Au group produced a significantly higher amount of ROS, as confirmed by semiquantitative analysis. F,G) Flow cytometry analysis and semiquantitative analysis: The flow cytometry plots show the distribution of cells in different stages (Q1: necrotic cells, Q2: late apoptotic cells, Q3: early apoptotic cells, Q4: normal cells). Compared with the Control group, the proportion of normal cells in the T‐rGO@Au group was significantly reduced, while the proportion of late apoptotic cells significantly increased, indicating that the T‐rGO@Au sample induces strong photothermal‐mediated tumor cell apoptosis.

To explore the photothermal effect of the scaffolds on K7M2‐wt cells in vitro, the calcein‐AM/PI double staining method was employed to observe the photothermal destructive effect of the scaffold on K7M2‐wt cells. As shown in **Figure** **7**A, K7M2‐wt cells exhibited almost no cell death without photothermal treatment, with an even distribution of cells across all groups. After photothermal treatment, the cells in the control and PTCP groups remained in good condition, indicating that short‐term mild heating had no significant destructive effect on the tumor cells. However, both T‐rGO and T‐rGO@Au groups exhibited excellent tumor‐inhibitory effects. After the photothermal treatment, the visual field was dominated by red‐stained cells, with almost no viable tumor cells. The semiquantitative analysis of the 10‐fold field of view showed the same trend: both the T‐rGO and T‐rGO@Au groups exhibited strong tumor‐eliminating effects (Figure 7B). The CCK‐8 method was used to quantitatively analyze the photothermal inhibitory effect of the scaffold on K7M2‐wt cells. Without the photothermal treatment, no difference in cell viability was observed among the groups (Figure 7C). After the photothermal treatment, the viability of the K7M2‐wt cells in the control and PTCP groups did not significantly change, indicating that they had no significant effect on tumor cell viability. However, the absorbance of the T‐rGO group significantly decreased, representing a decrease in cell vitality and indicating effective damage to K7M2‐wt cells. The photothermal efficacy of the T‐rGO@Au group was the highest, leading to a statistically significant decrease in K7M2‐wt cell viability compared to other groups. The excellent antitumor effect of the T‐rGO@Au scaffold stems from NIR excitation, which enables rGO and AuNP to exert a destructive effect on tumor cells.

The potential tumor elimination mechanism of T‐rGO and T‐rGO@Au scaffolds was further investigated by detecting reactive oxygen species (ROS) in tumor cells of each group. As shown in Figure 7D,E, before photothermal treatment, no significant ROS induction was observed in K7M2‐wt cells for the control, PTCP, T‐rGO, and T‐rGO@Au groups. After the photothermal treatment, the K7M2‐wt cells in the PTCP, T‐rGO, and T‐rGO@Au groups afforded different degrees of ROS, while those in the control group afforded a very small amount of ROS, which can be ignored. After photothermal treatment, only sporadic cells in the PTCP group exhibited ROS generation, which could be related to the above mentioned photothermal effect of TCP. In the T‐rGO group, a small number of cells produced ROS, which was more than that produced by the cells in the TCP group. However, the K7M2‐wt cells in the T‐rGO@Au group produced considerable ROS after photothermal treatment, which was more than that produced by the cells in the T‐rGO group, and the fluorescence intensity was also higher. Therefore, the photothermal destructive mechanism of the T‐rGO and T‐rGO@Au groups on K7M2‐wt cells could be related to the generation of intracellular ROS, which aligns with the findings of previous studies.^[^ ^44^ ^]^

To further investigate the mechanism of cell damage, flow cytometry was employed to classify the status of tumor cells following photothermal treatment. As shown in Figure 7F,G, the T‐rGO and T‐rGO@Au groups exhibited a significantly higher number of necrotic cells compared to other groups. The T‐rGO@Au group had the highest proportion of late apoptotic cells (over 60%), followed by T‐rGO. Additionally, the T‐rGO@Au group showed the lowest proportion of normal cells, with a statistically significant difference compared to all other groups, followed by the T‐rGO group. These findings suggest that the T‐rGO and T‐rGO@Au scaffolds induce tumor cell destruction primarily through the apoptosis pathway via photothermal therapy (PTT).

**Figure 8:**
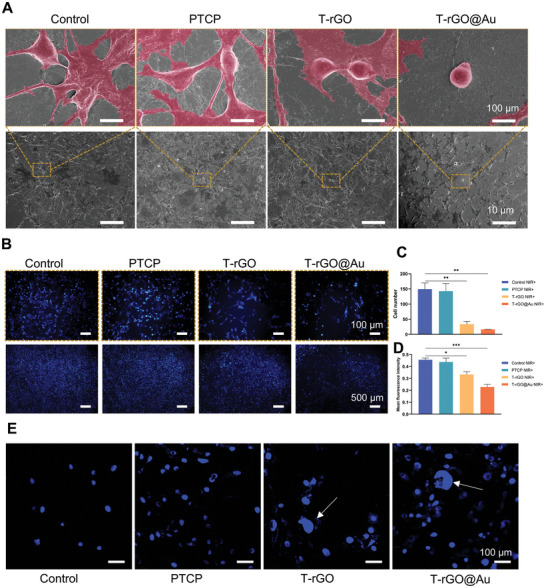
In vitro PTT antitumor assay. A) SEM images showing the effects of different samples on K7M2‐wt cells after photothermal treatment. The T‐rGO@Au group exhibited the most significant damage to cell morphology, with a marked decrease in the cytoskeleton. B–D) Hoechst staining and semiquantitative analysis: The fluorescence intensity of nuclear staining and the number of cells in the T‐rGO@Au group were significantly reduced, indicating severe nuclear damage. E) Confocal microscopy images showing nuclear damage in different sample groups after photothermal treatment. Arrows indicate prominent nuclear fragmentation in the T‐rGO and T‐rGO@Au groups.

To evaluate the influence of the photothermal effect of the scaffold on the tumor cell morphology, the morphology of K7M2‐wt cells after photothermal treatment was examined using an electron microscope (**Figure** **8**A). After the photothermal treatment, the K7M2‐wt cells in the control and PTCP groups still maintained normal morphology, displaying a spindle shape with protruding pseudopodia. This denotes that the photothermal treatment did not influence the tumor cells. In the T‐rGO group, after the photothermal treatment, the number of K7M2‐wt cells decreased and the cytoplasm condensed. The T‐rGO@Au group displayed the best photothermal effect, the number of K7M2‐wt cells significantly decreased, the proportion of OS cells with concentrated cytoplasm increased, and spherical cells appeared. Through PTT, the T‐rGO@Au group exhibited the most pronounced effect on the morphological destruction of tumor cells, which is indicative of tumor cell elimination.

After determining the effect of photothermal treatment on the OS cell morphology, the destruction of the tumor nucleus was investigated via Hoechst staining. As highlighted in Figure 8B,D, the number of nuclei in the T‐rGO and T‐rGO@Au groups decreased under NIR light, which was due to the decrease in cell adhesion because of the destruction of K7M2‐wt cells. This indirectly reflects the damage to the tumor cell function caused by PTT in T‐rGO and T‐rGO@Au groups. Nucleus morphology was also observed using a confocal laser microscope. As shown in Figure 8E, after PTT, the control and PTCP groups showed normal round or mitotic nuclear morphologies, while nucleoplasm condensation occurred in the T‐rGO group. In the T‐rGO@Au group, nuclear and cytoplasmic condensation as well as nuclear morphological destruction were observed after PTT. These results suggest that PTT in T‐rGO and T‐rGO@Au groups can effectively destroy the nuclear structure of OS cells.

Through transcriptome sequencing and bioinformatics analysis, the mechanism underlying tumor cell damage induced by T‐rGO@Au PTT was investigated. Totally, 2153 differentially expressed genes were observed, of which 1096 were upregulated and 1057 were downregulated (Figures S6A and S6B, Supporting Information). GO analysis showed that T‐rGO@Au mainly influenced key biological processes, including translation, nucleosome assembly, transcription, and cell cycle, suggesting that its photothermal effect could damage the DNA in tumor cells by interfering with the transcription and translation processes (Figure S6C, Supporting Information). KEGG analysis demonstrated that T‐rGO@Au regulates ribosome function, apoptosis, base repair, and related cellular processes through MAPK and Rap‐1 signaling pathways, thereby promoting tumor cell death (Figure S6D, Supporting Information). Moreover, PPI network analysis revealed (Figure S6E, Supporting Information) that the photothermal effect coordinately regulates mechanisms such as apoptosis, necrosis, and mitophagy through multiple key genes (e.g., Jun, Mapk14, and Casp3) and signaling pathways (including MAPK and Rap‐1).

### 3.7 In Vivo Antitumor Essay

**Figure 9:**
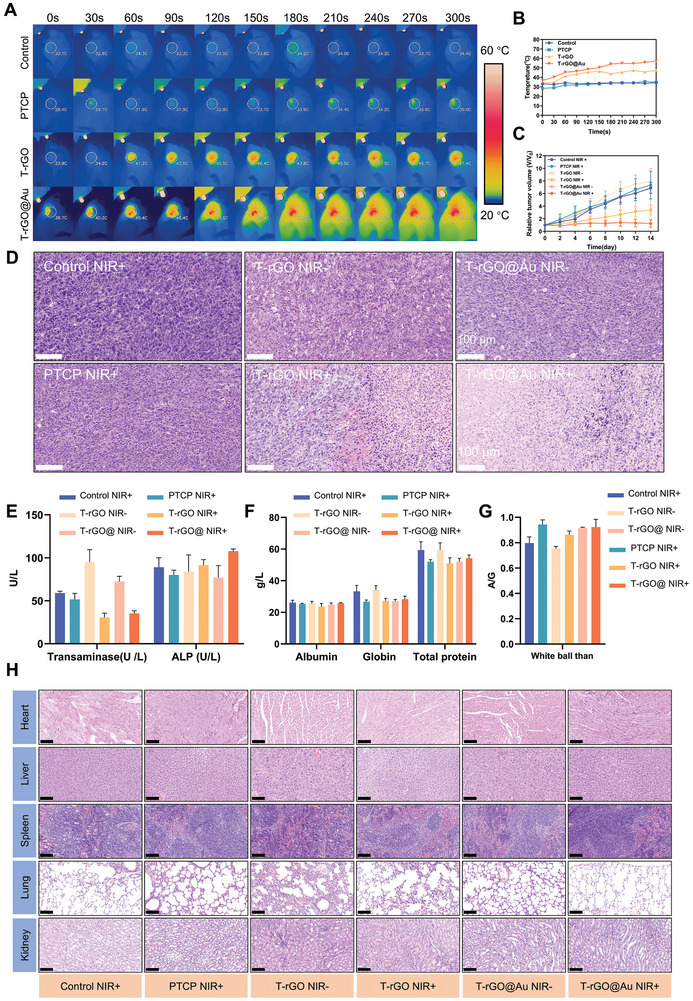
In vivo photothermal antitumor experiment. A,B) In vivo NIR thermal imaging and temperature‐time variation curves in mice. After 300 s of NIR irradiation, the temperature at the tumor site in the T‐rGO@Au group increased to ≈60 °C, significantly higher than that in the PTCP (≈45 °C) and T‐rGO (≈50 °C) groups. In contrast, the temperature in the control group only increased to about 32 °C, demonstrating the superior photothermal conversion performance of the T‐rGO@Au group. C) Growth curve of tumor volume in each group after 14 days of photothermal treatment. The T‐rGO@Au group showed a significant inhibition of tumor growth under NIR+ conditions, highlighting its excellent antitumor effect. D) H&E staining of tumor tissues showed large areas of necrosis in the T‐rGO@Au NIR+ group, while the tumor structures in other groups remained relatively intact, and cell morphology appeared normal. E–G) Effects of photothermal treatment on hematological indicators in mice. Aminotransferase (ALT/AST) and alkaline phosphatase (ALP) levels did not significantly differ among the groups, suggesting that liver function was not significantly affected. F) Albumin, globulin, and total protein levels did not significantly vary among the groups, indicating normal protein metabolism. G) Albumin/globulin (A/G) ratio showed no significant abnormalities among the groups, further supporting the safety of the treatment. H) H&E staining of major organs (heart, liver, spleen, lung, and kidney) of mice from each group 14 days after treatment. No obvious pathological changes were observed in any of the organs, indicating that PTT did not cause significant toxicity to the major organs. Scale bars, 100 µm.

In the in vivo antitumor experiment, the photothermal temperature curve of all the groups was obtained by recording the temperature at different times (**Figure** **9**A,B). The control group did not exhibit photothermal effects, and the temperature remained at 34.4 °C after photothermal treatment. The PTCP group displayed the same trend as the control group, with the temperature slightly increasing and stabilizing at 35.0 °C. This limited temperature is insufficient to destroy tumor tissue. In the T‐rGO group, the temperature quickly increased, but the maximum temperature only remained at 45–47 °C. After AuNP addition, the T‐rGO@Au group temperature increased to 50 °C within 150 s and finally stabilized at around 57.3 °C, demonstrating that the T‐rGO@Au scaffold can reach the temperature required to exert its antitumor function. In the i*n vivo* environment, the T‐rGO@Au scaffold still displayed excellent photothermal capabilities, which mainly stems from the synergistic photothermal effect of rGO and AuNP. The above results show that the T‐rGO and T‐rGO@Au scaffolds afford good photothermal effect in vivo and that the photothermal effect of the T‐rGO@Au scaffold is the best, which can be maintained above 50 °C. PTT relies on temperature, and NIR's strong tissue penetration allows for the effective ablation of deep tumor tissues.

As displayed in Figure 9C, the tumor volume for each group was recorded every 2 days until 14 days. The tumor volume of the T‐rGO@Au scaffold did not increase after NIR irradiation, indicating that it has a good inhibitory effect on tumor growth. However, the tumors in the non‐NIR‐irradiated group showed a normal growth trend; thus, the antitumor effect of the T‐rGO@Au scaffold stemmed from the photothermal effect. Similarly, the T‐rGO scaffold without NIR irradiation showed no inhibitory effect on tumor growth. However, NIR irradiation limited the tumor volume growth of the T‐rGO scaffold, displaying a slow growth trend. The T‐rGO scaffold somewhat inhibited the tumor growth, but the effect was not as good as the T‐rGO@Au scaffold. Moreover, the control and PTCP groups displayed no significant therapeutic effects even under NIR irradiation. On the 14th day after the operation, the tumor tissues were stripped from the mice and photographed. As depicted in Figure S7B (Supporting Information), among all the NIR‐irradiated groups, T‐rGO@Au NIR+ afforded the smallest tumor volume, followed by T‐rGO NIR+. The tumor size was significantly smaller for T‐rGO@Au NIR+ compared to that for T‐rGO@Au NIR−, and similar behavior was observed for the T‐rGO scaffolds.

Figure 9D displays the H&E staining images of the tumor tissues from each group. The cytoplasm and nucleus of the tumor cells in the control NIR+, PTCP NIR+, T‐rGO NIR− and T‐rGO@Au NIR− groups were clear and intact. The tumor cells in the T‐rGO NIR+ group partially lost their normal spindle cell structure, and the cell nucleus (purple) was destroyed, but some tumor cells still exhibited relatively complete cell and cell nuclear structures. The tumor cells in the T‐rGO@Au NIR+ group lost their basic cell arrangement, most nuclei were destroyed, some nuclei were severely fragmented, and the cell morphology changed into a spherical shape, reflecting serious damage to the tumor tissue and excellent therapeutic effect. The H&E staining results further substantiated the most effective PTT treatment with T‐rGO@Au NIR+.

Assessing the toxicity of adjacent areas treated with PT is particularly important for stent compatibility testing. Therefore, H&E section staining was performed on the mice skin tissues after one cycle of PTT to evaluate the influence of PTT on the tissue structure, interstitial components, and cell morphology of the surrounding skin. As shown in Figure S7A (Supporting Information), the control and PTCP groups displayed normal skin tissue structures due to low photothermal temperatures. For the T‐rGO scaffold, since the temperature of the photothermal effect in the body was maintained below 50 °C, the burns to the surrounding skin tissue were relatively mild. Owing to its high body temperature (57 °C) and ROS induction ability, the T‐rGO@Au scaffold displayed the best tumor inhibition effect in the in vivo environment, which inevitably led to potential toxicity to the surrounding skin. The H&E results showed that the T‐rGO@Au group caused burns in the surrounding skin and the accumulation of local inflammatory cells but did not exhibit necrosis in the surrounding tissue, as seen in the tumor tissue. This indicates that the T‐rGO@Au scaffold has a strong destructive effect on tumor cells while imparting minimal toxicity to the surrounding tissues. After sampling, the H&E staining images of the lung, liver, spleen, kidney, and heart of the mice for each group were obtained (Figure 9H). The results showed that the main organ cells of all groups grew well, and the cytoplasm and nucleus were clearly arranged and intact. Furthermore, the implants in each group had no obvious toxicity and destructive effect on the viscera within 14 days. Three blood samples from each group were also obtained. Figure 9E,G shows that the blood biochemical indices (albumin, total protein, globulin, albumin/globulin ratio, transaminases, and ALP) were within normal ranges, confirming the biological safety of the implants in all groups.

## 4 Conclusion

To specifically address the requirements for treating OS, this study aimed to design and construct a T‐rGO@Au scaffold with dual functionality, exhibiting both excellent osteogenic properties and antitumor capabilities. Achieved sustained and controllable tumor‐destructive capability. Based on the “antenna effect” of AuNPs, T‐rGO@Au exhibited high photothermal conversion efficiency. The results showed that under NIR excitation, the T‐rGO@Au scaffold induced tumor cell damage through signaling pathways, such as MAPK and Rap‐1. The nucleus of tumor cells was damaged, the cell translation and transcription function were impaired, and the cell morphology was damaged. The T‐rGO@Au scaffold resulted in tumor cell apoptosis and necrosis, effectively damaging tumor cells in vitro and disrupting tumor tissue in vivo. Moreover, to repair bone defects, a PTCP scaffold with cancellous bone elastic modulus was prepared via 3D printing, and the rGO@AuNPs coating was loaded on the scaffold surface. The T‐rGO@Au scaffold exhibited excellent biocompatibility and osteogenesis. It also promoted the early and late osteogenic differentiation of MC3T3‐E1 cells. In vivo experimental results showed that at 12 weeks, the T‐rGO@Au group afforded the highest BV/TV, and the new bone was tightly integrated with the scaffold. Overall, the T‐rGO@Au scaffold with antitumor and osteogenic functions can be used to effectively treat bone defects after OS surgery and has ideal clinical translation prospects.

## Conflict of Interest

The authors declare no conflict of interest.

## Supporting information



Supporting Information

## Data Availability

The data that support the findings of this study are available from the corresponding author upon reasonable request.
