# Supplementary material for: “Antenna Effect”‐Enhanced AuNPs@rGO Photothermal Coating Promotes 3D Printing of Osteogenic Active Scaffolds to Repair Bone Defects after Malignant Tumor Surgery
Source: Adv Sci (Weinh). 2025 Feb 20;12(15):2417346. doi: 10.1002/advs.202417346 (PMC12005794; doi:10.1002/advs.202417346)
Supplement: Supplementary file 1 — Supporting Information [file ADVS-12-2417346-s001.docx]

**Supporting Information**


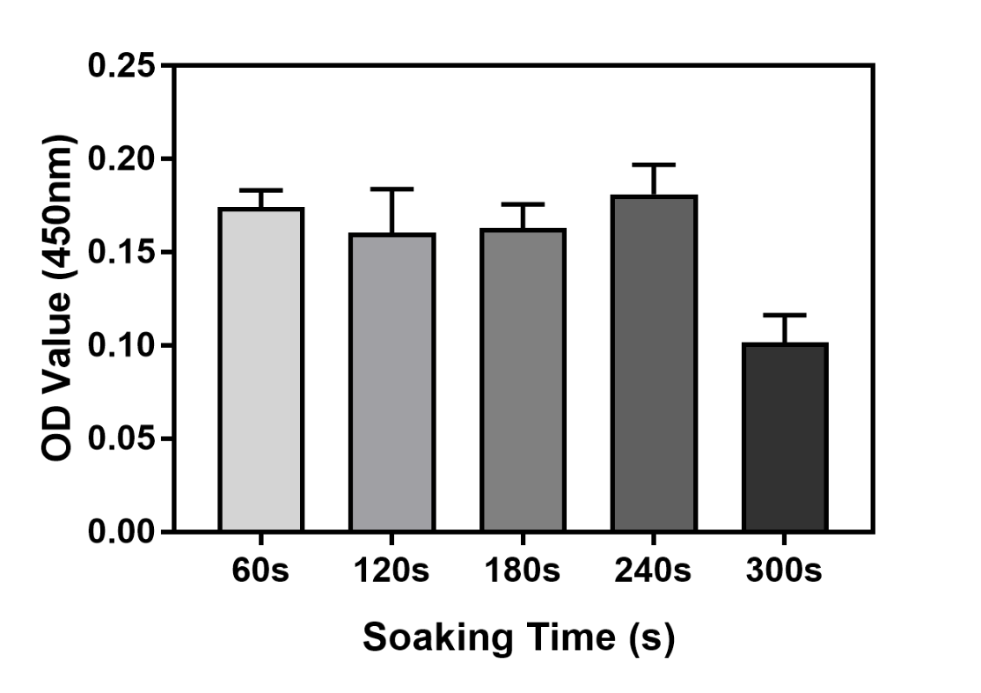


**Figure S1.** The modification time of rGO-AuNP was screened through cell viability experiments.


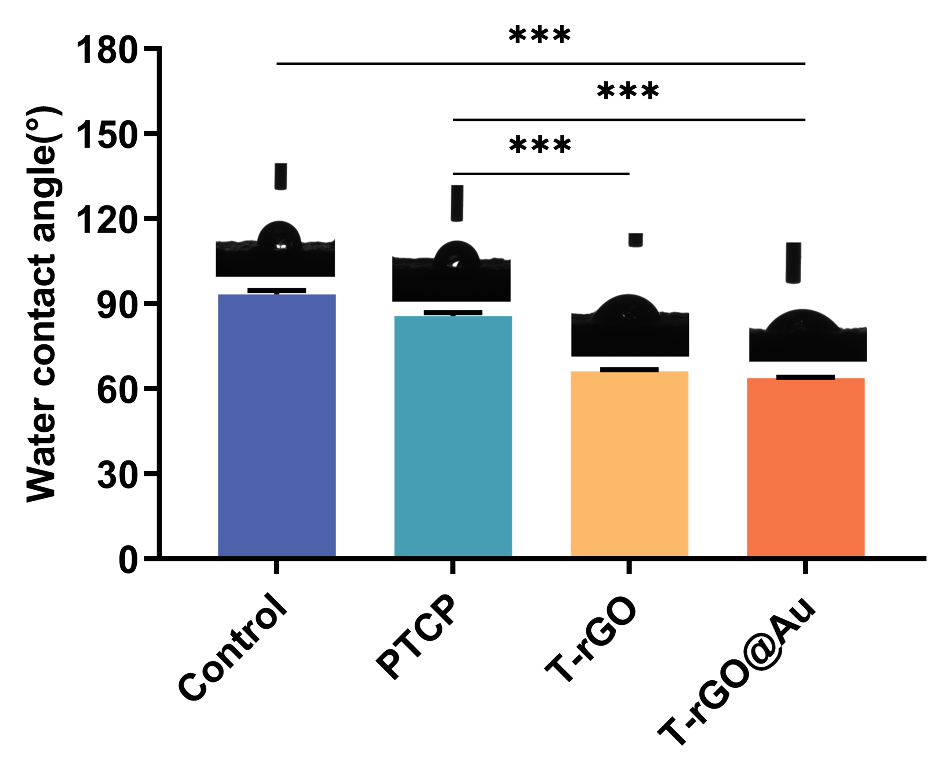


**Figure S2.** Water contact angle of the Control, PTCP, T-rGO, and T-rGO@Au samples. The T-rGO@Au group has the lowest water contact angle, shows the most significant change in surface hydrophilicity, and is significantly different from other groups.


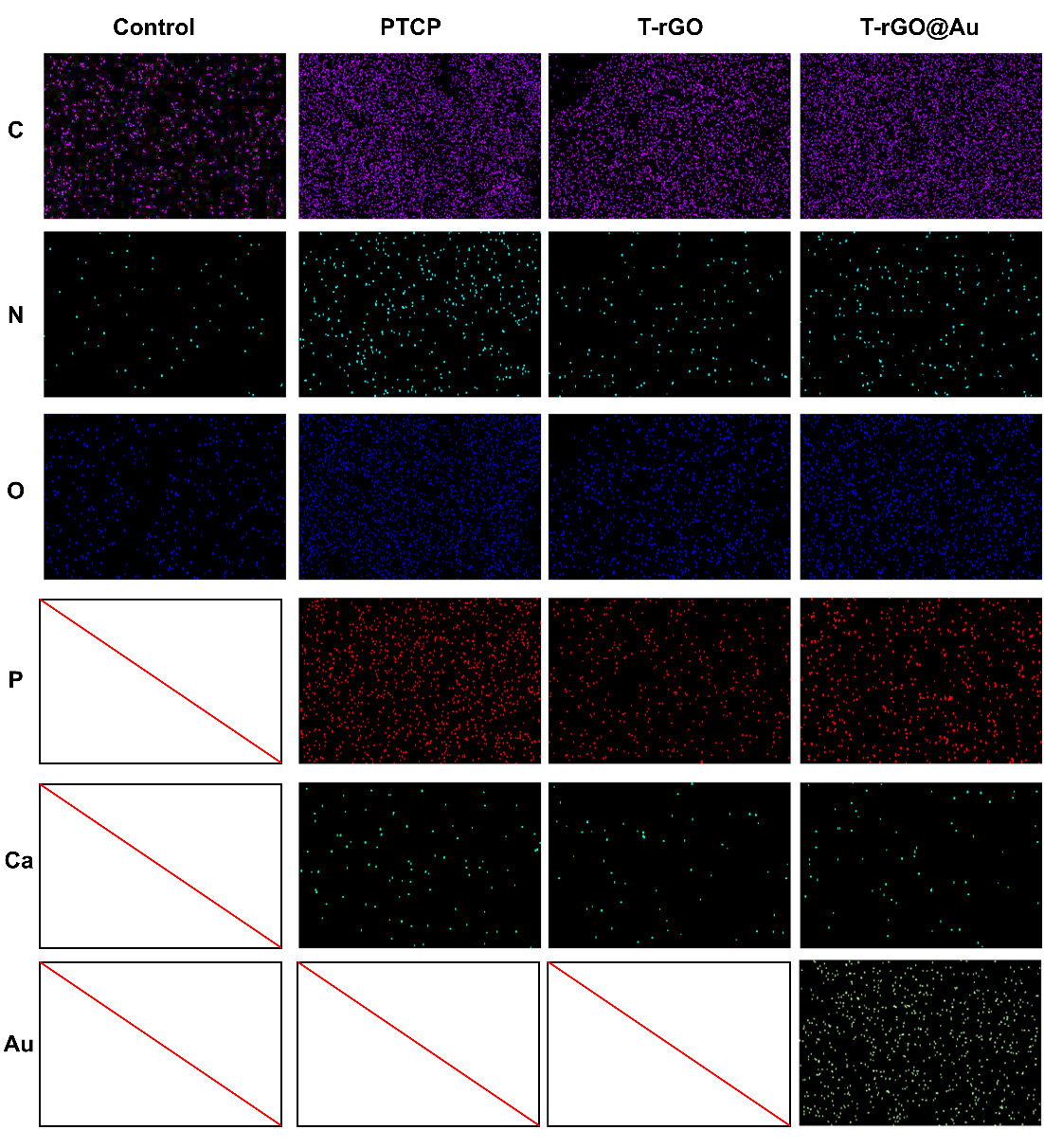


**Figure S3.** EDS spectra of each group of scaffolds. The element distribution diagram showed that the Control group had no specific elements, the PTCP group was rich in N and P, and the T-rGO@Au group showed the presence of Au.


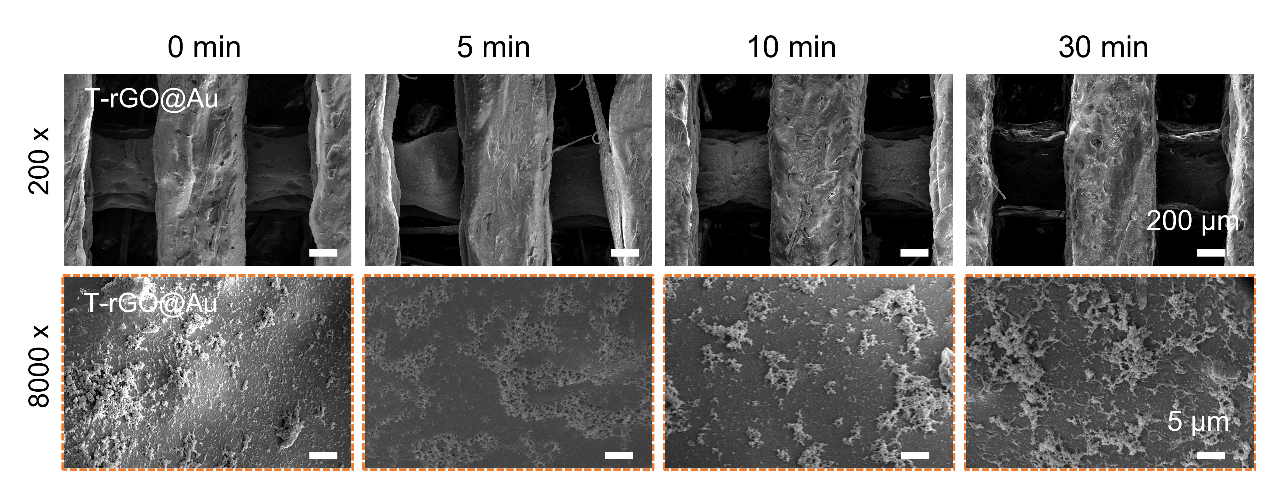


**Figure S4.** Surface SEM images of the T-rGO@Au group scaffolds after ultrasound treatment for different times. The SEM images of the T-rGO@Au scaffold surface under different ultrasonic treatment times showed that although the ultrasonic treatment time increased, the PDA structure adhered to the scaffold surface remained stable and no obvious peeling or damage was observed.


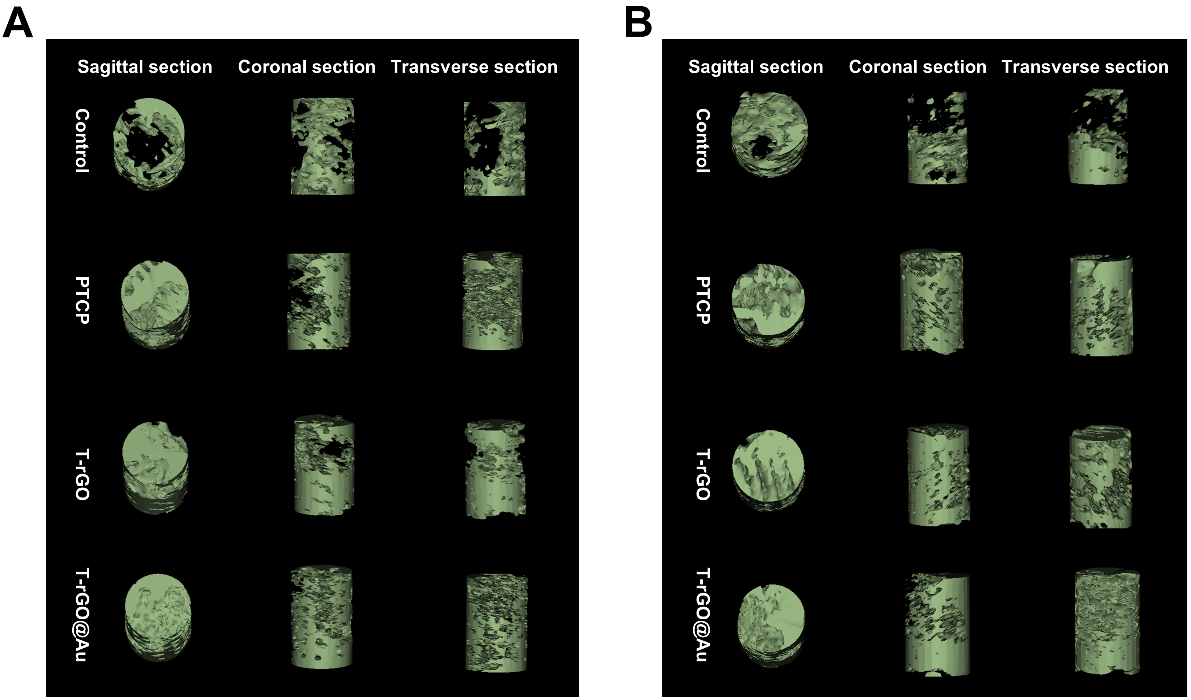


**Figure S5.** 4 (A) and 12 (B) weeks after the implantation of Control, PTCP, T-rGO and T-rGO@Au scaffolds, Micro-CT three-dimensional images of the new bone were obtained. Micro-CT three-dimensional imaging shows that after 4 weeks (A) and 12 weeks (B) of implantation, the T-rGO@Au group has the most significant new bone formation, showing the best bone repair effect compared with other groups.


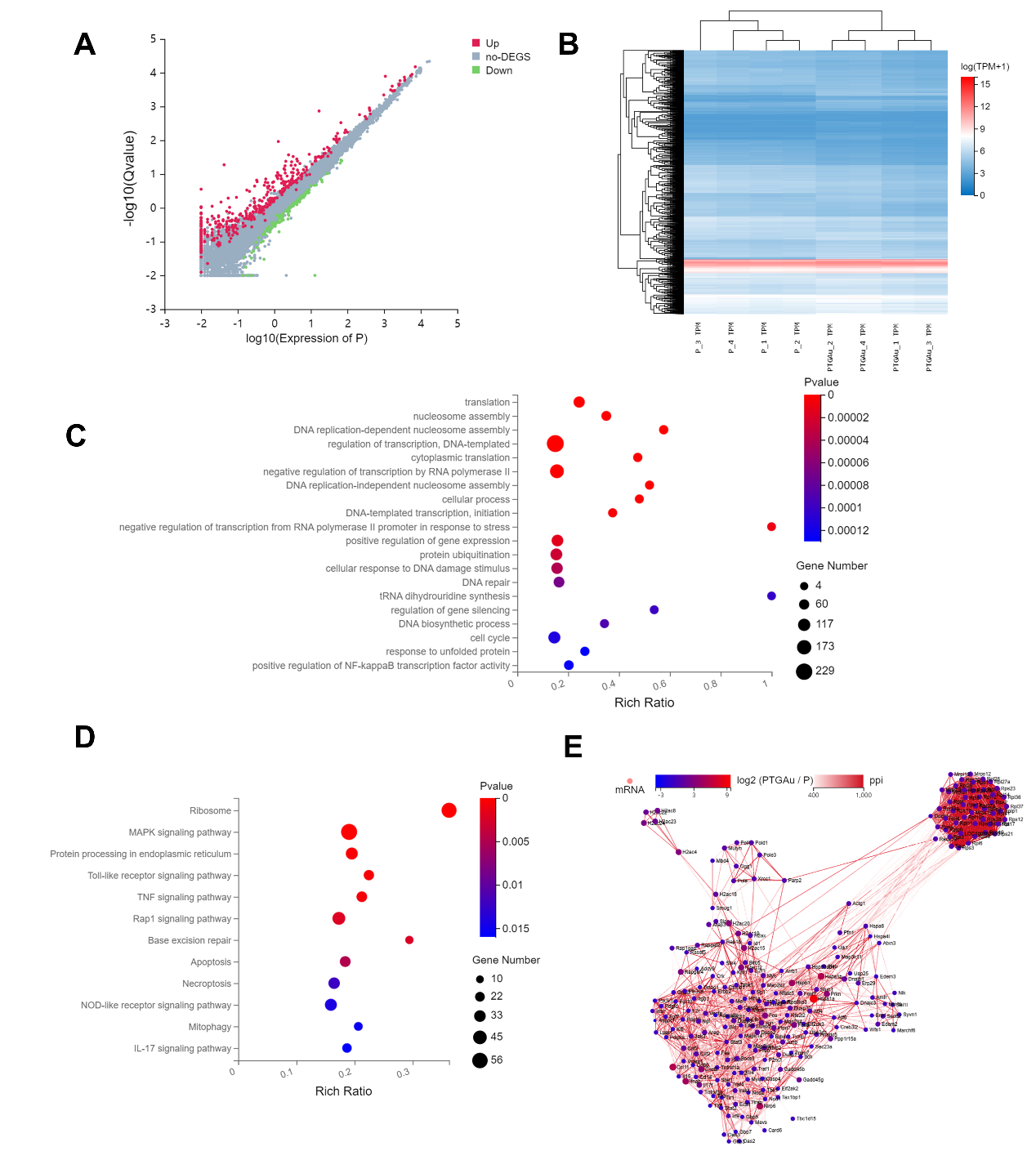


**Figure S6.** RNA-seq and bioinformatics analysis. (A) Scatter plot of differentially expressed genes. (B) cluster heat map of differentially expressed genes. (C) GO analysis of the biological process. (D) KEGG ontology analysis. (e) PPI interaction network analysis.


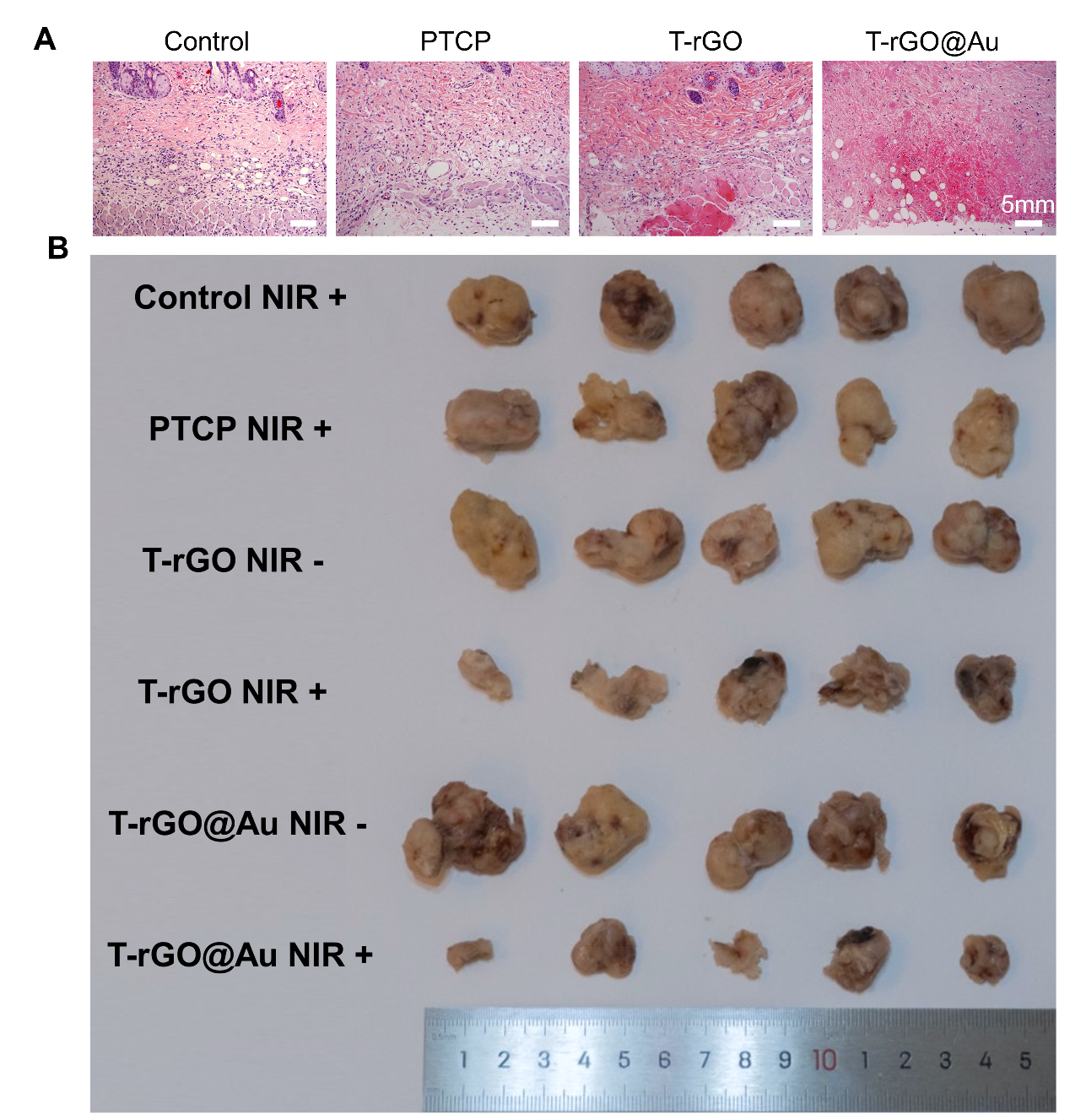


**Figure S7.** (A) HE staining results showed that when the photothermal treatment reached 55°C, the surrounding skin tissue of the T-rGO@Au group showed signs of slight burns, but the overall degree of damage was low and the inflammation was mild, indicating that the effect of its photothermal treatment on the surrounding normal tissues was controllable. (B) In the tumor HE section image, the tumor volume in the T-rGO@Au NIR+ group was significantly reduced, indicating that it can effectively eliminate tumors while causing limited thermal damage to normal tissues and has high treatment safety.

| **Parameters name** | **PEI** | **PTCP** |
| --- | --- | --- |
| Temperature of zone-1 | 280-300℃ | 280-300℃ |
| Temperature of zone-2 | 300-320℃ | 300-320℃ |
| Temperature of zone-3 | 310-330℃ | 310-330℃ |
| Diameter of lager head | 30 mm | 30 mm |
| Diameter of smaller head | 10 mm | 10 mm |
| Screw speed | 20-25 r/min | 20-25 r/min |
| Speed of tractor | 19 r/min | 19 r/min |
| Cavity pressure | 1.8 Mpa | 1.8 Mpa |

**Table S1.** Parameter setting of twin-screw extruder extrusion.

| Gene | Primer sequence | Product size  (bp) |
| --- | --- | --- |
| OCN | F:5'GGACCATCTTTCTGCTCACTCT3' | 22 |
|  | R:5'CGGAGTCTGTTCACTACCTTATTG3' | 24 |
| RUNX-2 | F:5'GGACTGTGGTTACCGTCAT3' | 19 |
|  | R:5'GGAGGATTTGTGAAGACTGTT3' | 21 |
| OPN | F:5'GGATGAATCTGACGAATCTC3' | 20 |
|  | R:5'TGAAAGTGTCTGCTTGTGTACTA3' | 23 |
| β-actin | F:5'CCTCTATGCCAACACAGT3' | 18 |
|  | R:5'AGCCACCAATCCACACAG3' | 18 |

**Table S2.** qPCR primer sequence.

| 4D | β-actin | OPN | RUNX-2 | OCN | 7D | β-actin | OPN | RUNX-2 | OCN |
| --- | --- | --- | --- | --- | --- | --- | --- | --- | --- |
| A | 23.83 | 30.02 | 33.62 | 36.02 | A | 22.60 | 30.26 | 31.79 | 33.93 |
|  | 22.82 | 30.83 | 33.51 | 35.20 |  | 22.59 | 31.51 | 31.55 | 34.20 |
|  | 23.77 | 31.92 | 34.04 | 35.77 |  | 22.98 | 32.08 | 33.14 | 35.90 |
| B | 23.61 | 29.96 | 33.82 | 35.60 | B | 22.02 | 30.34 | 31.26 | 34.57 |
|  | 23.66 | 30.39 | 33.02 | 35.94 |  | 22.52 | 30.61 | 32.30 | 34.12 |
|  | 23.04 | 31.01 | 33.91 | 34.60 |  | 22.34 | 30.18 | 30.68 | 33.71 |
| C | 24.96 | 30.50 | 34.98 | 36.10 | C | 22.81 | 30.03 | 32.10 | 33.81 |
|  | 22.47 | 28.58 | 32.09 | 35.20 |  | 23.53 | 31.60 | 32.03 | 32.92 |
|  | 24.25 | 29.84 | 33.21 | 36.07 |  | 24.38 | 31.43 | 32.40 | 34.06 |
| D | 23.88 | 30.63 | 33.47 | 36.74 | D | 23.47 | 30.30 | 31.58 | 33.35 |
|  | 24.56 | 30.30 | 33.89 | 37.07 |  | 23.18 | 30.08 | 31.30 | 32.64 |
|  | 24.42 | 30.64 | 35.52 | 35.00 |  | 23.15 | 30.35 | 31.64 | 33.08 |

**Table S3.** qPCR CT value data at 4 days and 7 days. A: Control group; B: PTCP group; C: T-rGO group; D: T-rGO@Au group.
